# WeChat-Delivered Mobile Medical Nutrition Therapy Intervention in Gestational Diabetes Mellitus: Randomized Controlled Trial

**DOI:** 10.2196/67410

**Published:** 2026-06-11

**Authors:** XiaoRong Wang, MiaoMiao Bian, Fang Li, Chang Zhou, LiNing Mu

**Affiliations:** 1 Community Healthcare Center, Jinan Central Hospital Affiliated to Shandong First Medical University, Jinan, Shandong, China; 2 Cardiovascular Department, Jinan Central Hospital Affiliated to Shandong First Medical University, Jinan, Shandong, China; 3 Graduate School, Shandong First Medical University, Jinan, Shandong, China; 4 School of Nursing and Rehablitation, Shandong University, Jinan, Shandong, China; 5 Diabetes Nursing Specialist Clinic, Jinan Central Hospital Affiliated to Shandong First Medical University, 105 Jiefang Road, Lixia District, Jinan, Shandong, 250013, China, 86 15318816507

**Keywords:** gestational diabetes mellitus, medical nutrition therapy, behavior change techniques, gestational weight gain, mobile health, mHealth

## Abstract

**Background:**

Medical nutrition therapy (MNT) serves as the foundational intervention in the clinical management of gestational diabetes mellitus (GDM) management. However, inadequate supportive care often hinders patients’ ability to sustain dietary modifications and self-management behaviors, particularly for complex regimens. Flexible online interventions are thus gaining interest as adjuncts to clinical care, with the potential to improve the outcomes of GDM self-management.

**Objective:**

This study sought to evaluate the benefits of WeChat-delivered medical nutrition therapy (WeMNT), a WeChat-delivered MNT intervention, for patients with GDM.

**Methods:**

A parallel-group, single-blind randomized controlled trial (with outcome assessor blinding) was implemented at a university hospital’s obstetric clinic. Eligible participants were those with a 24‐ to 28-week singleton pregnancy, a GDM diagnosis confirmed by the 75 g oral glucose tolerance test, no requirement for insulin therapy, and the ability to use a smartphone and WeChat, as well as to communicate in Chinese. Participants were randomized 1:1 to the intervention (n=47, 50%) or control group (n=47, 50%) using a random number table. The WeMNT intervention was designed per the behavior change wheel framework: (1) analyze capability-, opportunity-, and motivation-based barriers related to MNT adherence; (2) formulate targeted intervention functions; and (3) select evidence-based behavior change techniques. Three WeChat mini-programs served as the core delivery platform to implement the multifaceted intervention functions. The primary outcomes measured in this study included fasting blood glucose, 2-hour postprandial glucose (2hPG), hemoglobin A_1c_ (HbA_1c_), and gestational weight gain (GWG). The secondary outcomes assessed included obstetric complications and neonatal parameters. Intention-to-treat analysis using adjusted generalized linear mixed models was conducted to evaluate the effects of the intervention.

**Results:**

From March 2023 to October 2023, a total of 94 participants were enrolled in this study. Among the participants, 88 (93.62%) individuals (mean age 32.18, SD 5.04 years) successfully completed the study, 44 (46.81%) in the intervention group and 44 (46.81%) in the control group. Approximately 68.18% (30/44) of the participants in the intervention group demonstrated sustained adherence, as expected, adhered to the diet plan ≥60% of the days. Compared with the control group, the intervention group showed a significantly greater decrease in GWG over time (group×time interaction: β=−1.96, 95% CI −3.58 to −0.34; *P*=.02), with no significant effects on fasting blood glucose. Conversely, significant decreases in 2hPG (β=−0.12, 95% CI −0.19 to −0.04; *P*<.001) and HbA_1c_ (β=−0.49, 95% CI −0.74 to −0.23; *P*<.001) were noted in the intervention group. No unexpected maternal adverse events occurred, and no significant effects on other health outcomes were detected.

**Conclusions:**

As a WeChat-delivered, behavior change wheel–informed intervention, WeMNT successfully reduced 2hPG, HbA_1c_, and GWG in GDM management. Its integrated self-management model yields robust evidence, validating this patient-centered tool as a viable option for clinical adoption.

## Introduction

### Background

Gestational diabetes mellitus (GDM) refers to a prevalent pregnancy-associated metabolic disorder, defined by the onset or initial identification of glucose intolerance during gestation [1]. According to the International Diabetes Federation, the age-standardized prevalence of GDM in low- and middle-income countries increased from 6.8% in 2011 to 8.8% in 2021, representing a weighted relative increase of 29.4% (95% CI 22.1%‐37.2%) after adjusting for diagnostic criteria and study heterogeneity, in parallel with the growing epidemic of obesity [2,3]. Suboptimal glycemic regulation during GDM is associated with increased maternal risks, including preeclampsia, cesarean delivery, and postpartum hemorrhage. Beyond the perinatal period, women with a history of GDM face an increased likelihood of developing chronic conditions such as type 2 diabetes, metabolic syndrome, and cardiovascular disease in the long run [4]. Offspring exposed to maternal hyperglycemia face immediate risks of macrosomia, birth trauma, neonatal hypoglycemia, hyperbilirubinemia, and polycythemia. These infants also demonstrate increased susceptibility to obesity and diabetes later in life.

Medical nutrition therapy (MNT) is the primary approach for managing GDM and is frequently effective in achieving desired blood glucose levels [5]. The significant benefits of dietary interventions in GDM management also include improved maternal outcomes and reduced neonatal macrosomia risk compared with conventional care [6]. Specifically, low–glycemic index (GI) diets have been shown to attenuate glycemic excursions while mitigating the need for pharmacological interventions [6,7]. These outcomes are hypothesized to result from delayed carbohydrate absorption and enhanced satiety signaling associated with low-GI macronutrient profiles [7]. However, controversy persists regarding optimal carbohydrate modulation strategies. While certain trials advocate restrictive carbohydrate regimens (≤40% of total energy intake) for acute glycemic reduction, emerging data suggest that liberal intake of high-fiber complex carbohydrates (eg, whole grains and legumes) may offer superior long-term metabolic benefits [8]. This paradigm prioritizes glycemic stability and preserves maternal nutritional adequacy, potentially reducing the risk of ketogenesis compared with severe caloric restriction [9]. Regardless of whether a restrictive or more liberal carbohydrate regimen is adopted, individualized nutritional plans that consider mothers’ specific characteristics (eg, health status, weight, ethnicity, and cultural preferences) are crucial for effective GDM management [10]. A joint care team comprising endocrinologists, obstetricians, certified dietitians, and diabetes education specialists is recommended to develop individualized treatment plans [5].

Enhanced dietary support significantly improved carbohydrate adherence in high-risk pregnancies [11]. Among obese women (BMI ≥30 kg/m²), those receiving weekly 90-minute dietitian-led group counseling on carbohydrate-controlled diets (40%‐45% of total calories) demonstrated a 47% reduction in days with excessive carbohydrate intake (≥10% above target) compared with controls over 24 weeks (adjusted odds ratio 0.53, 95% CI 0.39‐0.72; *P*<.001) [11]. A dietary plan is key to managing GDM, yet women often struggle to adopt and sustain new dietary habits and self-management behaviors [12]. Long-term adherence to complex dietary plans is challenging without continuous support [13]. Providing adequate self-management support empowers patients to control their diet and blood glucose levels. As they gain self-management skills, positive behavioral changes become evident [13]. Prescribed treatment plans instill a strong sense of responsibility in women with GDM, motivating them to strictly adhere to dietary and self-management guidelines [12]. Nevertheless, their clinical appointments with health care professionals are frequently too brief to provide the in-depth, comprehensive guidance necessary for effective GDM management [14,15]. Mobile health (mHealth) and digital interventions have become essential complementary tools for supporting women with GDM and health care professionals. Recent studies report an increasing reliance on digital platforms because of their flexibility and convenience, which help overcome limited clinical contact time and provide continuous, personalized support for self-management [16,17]. Both women and health care professionals expressed openness to using technology as an adjunct to standard care but emphasized the need for improvements in usability, such as enhanced content layout, user interface design, and data visualization [17]. Participants also highlighted the importance of features supporting diverse data recording, personalization, comprehensive information delivery, and effective communication with health care providers and peer networks [17]. Currently, various mHealth tools often do not fully address the needs of women with GDM [18]. Both patients and health care professionals have stressed the importance of improving usability, personalization, information adequacy, and communication functionalities [18]. These findings highlight the ongoing need to develop and optimize mHealth solutions to better support self-management among women with GDM.

### Theoretical Framework

According to the literature, current behavior change interventions often have 2 major limitations: insufficient theoretical support and underuse of evidence-based frameworks [19]. To address these limitations, we adopted the behavior change wheel (BCW) as our core theoretical foundation [19]. With the embedded capability, opportunity, motivation behavior (COM-B) model, the BCW is distinguished in supporting researchers to analyze the origins of behavioral barriers and design theory-informed interventions [19]. Specifically, capability refers to an individual’s psychological (eg, knowledge and cognitive skills) or physical (eg, motor ability) capacity to perform the target behavior; opportunity encompasses external enablers or constraints, including the physical environment (eg, access to resources) and social support (eg, family encouragement); and motivation integrates conscious decision-making (eg, goal setting) and subconscious drives (eg, emotional attitudes) that influence behavioral intent [19]. In the system, COM-B elements interact dynamically to shape behavior, enabling designers to first identify context-specific barriers to the target behavior and then map them to targeted intervention functions most likely to drive meaningful change. This systematic mapping, in turn, facilitates the precise translation of intervention functions into fine-grained, measurable, and replicable behavior change techniques (BCTs)—a critical step in ensuring the scientific rigor and scalability of the intervention [19]. By grounding the intervention in the BCW, we establish a transparent, theory-driven link between identified behavioral barriers, intervention design choices, and measurable outcomes—addressing the field’s need for more structured, evidence-based approaches to behavior change intervention development.

### Goal of This Study

Building on the BCW framework, we developed WeChat-delivered medical nutrition therapy (WeMNT), a WeChat-delivered MNT program tailored for women with GDM. On the basis of clinical expertise and patient feedback, we hypothesized that WeMNT will improve the effectiveness of GDM management. This study aimed to evaluate the benefits of WeMNT, a WeChat-delivered MNT intervention program developed from clinical knowledge and the BCW framework, for women with GDM.

## Methods

### Study Design and Participants

This single-blind, parallel-group randomized controlled trial (RCT)—with blinding limited specifically to outcome assessors—was carried out at the obstetric clinic affiliated with a university hospital. The reporting of this study adhered to the CONSORT (Consolidated Standards of Reporting Trials) guidelines (Checklist 1). The inclusion criteria included singleton pregnancy at 24 to 28 weeks, GDM diagnosed by a 75 g oral glucose tolerance test (OGTT) [20], no insulin requirement, and the ability to use a smartphone with WeChat and to communicate in Chinese.

### Sample Size Calculation

The study was powered based on the primary outcome of hemoglobin A_1c_ (HbA_1c_). To determine the appropriate sample size, we first identified the effect size based on the relevant literature. A meta-analysis revealed that telemedicine interventions for women with GDM yielded an effect size of 0.63 (Cohen *d*, ie, a standardized mean difference between groups) for HbA_1c_ reduction; specifically, studies using WeChat as the intervention tool (6 RCTs in total) demonstrated a greater effect size of 0.84 for HbA_1c_ improvement [21]. Considering both clinical relevance and methodological conservatism, we determined a minimum effect size (Cohen *d*) of 0.65 for the sample size calculation.

G*Power 3.1 software [22] was used to perform the a priori sample size calculation using the following key parameters: effect size (*d*)=0.65, significance level (α)=.05 (2-tailed), and statistical power (1−β)=.80. The calculation results indicated a basic sample size of 78 participants. To address a potential 15% attrition rate, a common consideration in longitudinal clinical studies, the final sample size was adjusted to 94 participants, which ensured adequate statistical power for detecting the prespecified effect on the primary outcome (HbA_1c_).

### Recruitment and Randomization

Participants were enrolled during their standard antenatal care appointments. Clinic physicians and nursing staff assisted in recruitment by disseminating study information to potential participants who met the eligibility criteria and had recently completed the OGTT. Interested candidates underwent an initial eligibility assessment by the research team. A follow-up appointment was then scheduled at the clinic, typically within 1 week of the OGTT, to discuss the study in detail, obtain written informed consent, and initiate enrollment.

After providing informed consent, eligible participants were randomly assigned to either the intervention group (n=47, 50%) or the control group (n=47, 50%) in a 1:1 allocation ratio, with randomization sequences generated by a computer. To ensure allocation concealment, sequentially numbered, sealed, opaque envelopes were used. This process preserved the integrity of the randomization and minimized selection bias.

### Intervention

To achieve the study’s objective, we followed the 3-stage protocol specified in the BCW intervention design guide [19]. First, preliminary assessments and literature review were conducted, and the COM-B barriers impeding GDM patients’ adherence to a personalized MNT plan were analyzed. The identified distinct COM-related barriers to MNT adherence include the following:

- Capability: deficits in nutritional knowledge and inability to formulate personalized MNT plans, accurately weigh food portions, or maintain dietary records
- Opportunity: lack of household weighing tools, work-related time constraints for learning and implementing MNT, and insufficient social support
- Motivation: inadequate awareness of MNT’s clinical importance, concerns about disrupting daily routines, and anxieties regarding the potential impacts of MNT

Second, targeted intervention functions—including education, enablement, modeling, and environmental restructuring—were selected to address these COM-specific barriers. Finally, building on this foundation, evidence-based BCTs were further identified to operationalize the selected functions [23]. Specifically, a group of BCTs validated for type 2 diabetes self-management in the literature was adopted [24].

For delivery, WeChat mini-programs were prioritized as the core platform because of their high accessibility and user familiarity. Three WeChat mini-programs were designated as the core delivery platform to implement diverse intervention functions and facilitate participants’ acquisition of relevant knowledge and practical skills. A schematic overview outlines the BCW-informed intervention development, COM categories, barriers, BCTs used, and WeMNT intervention operationalization, including BCT embedded in mini-program features (Table 1).

**Table 1.** Intervention development: capability, opportunity, and motivation (COM) categories, barriers, behavior change techniques (BCTs), and mini-programs.

COM categories and key barriers	Intervention functions	Policy categories	BCTs	WeChat platform	Intervention contents
Capability					
Deficits in nutritional knowledge	Education	Communication	BCT4.1 Instruction on how to perform a behavior BCT 4.2 Information about antecedents	Mini-program “Diet Diary-Food Charts”	Via the program, women learn GI^a^ knowledge and practical nutritional skills (BCT4.1) and get nutritional choice antecedent info (BCT4.2).
Inability to accurately understand food portions and formulate personalized MNT^b^ plans	Training	Service provision	BCT4.1 Instruction on how to perform a behavior BCT6.1 Demonstration of the behavior BCT1.4 Action planning	Mini-program “Diet Diary”	Via the program, dietitians teach women to input clinical data, select food types and weights/portions, and make personal MNT plans (BCT4.1), demonstrate plan design and adjustment (BCT6.1), and cocreate daily meal schedules with reminders (BCT1.4).
Inability to maintain dietary records	Training	Service provision	BCT2.3 Self-monitoring of behavior	Mini-program “Diet Diary”	Women use the program to log daily food portion weights and receive alerts on whether intake meets, falls below, or exceeds targets.
Opportunity					
Lack of household weighing tools	Environmental restructuring	Service provision	BCT12.5 Adding objects to the environment		The research team provided digital food scales to support accurate measurement.
Work-related time constraints for implementing MNT	Environmental restructuring	Service provision	BCT7.1 Prompts/cues	Mini-program “Diet Diary”	Aligned with the MNT plan, the mini-program sends timed pop-up reminders (eg, “Check meal compliance 10 minutes premeal,” “Log daily diet 5 minutes before bedtime”).
Insufficient social support	Modeling	Service provision	BCT12.2 Restructuring the social environment	Group discussion	A nurse moderated a WeChat group for peer support; women shared experiences/strategies weekly.
Motivation					
Inadequate awareness of MNT’s clinical importance	Education	Service provision	BCT4.3 Information about health consequences BCT2.2 Feedback on behavior	Mini-program “Diet Diary”; Video call	Via the program, women self-learn MNT’s medical value, disease-prognosis links, and noncompliance risks to fill cognitive gaps (BCT4.3). Persistent glucose issues triggered video consultations and potential multidisciplinary referrals (BCT2.2).
Worries about MNT disrupting daily routines	Modeling	Service provision	BCT3.3 Social support	Group discussion	A nurse moderated a WeChat group for peer support; women shared experiences or strategies weekly.
Fears of MNT causing potential health impacts	Enablement	Service provision	BCT2.4 Self-monitoring of the outcomes BCT2.2 Feedback on behavior	Mini-program “Pregnancy Sugar Weight Record” and “Fetal Weight Assessor” Instant message or voice call	Women input blood glucose, body weight, and fetal weight into the programs, which generate linked trend curves for all 3 and alert if values meet, fall below, or exceed targets. Diabetes nurses weekly reviewed backend data, provided WeChat feedback, and adjusted meal plans with dietitians for off-target patients.

a GI: glycemic index.

b MNT: medical nutrition therapy.

### Intervention Features

In this mHealth study, we used well-established, publicly available, nonprofit WeChat mini-programs as the intervention platform. First, the “Diet Diary” mini-program was developed by a certified dietitian, Zhongyi Gu, as a nonprofit public health tool aligned with the American Diabetes Association guidelines for GDM [5]. This mini-program integrates personalized meal planning, real-time dietary tracking, and weekly professional feedback to facilitate effective dietary management for users. Dietitians leveraged the mini-program to deliver step-by-step instructions on MNT plan formulation, demonstrating both the plan design logic and mini-program operation through real-time interface walkthroughs to teach women how to use the platform independently. They then collaborated with women using the mini-program’s action planning module to develop structured daily meal plans that specified food types and weights for each meal, with embedded reminders and real-time adjustment features tailored to individual clinical needs and daily routines. On the basis of the mini-program’s “Food Charts” self-learning feature, women acquired knowledge about the GIs of 476 common foods, food selection, weighing, recording, and meal arrangement; access to antecedent information about nutritional choices; and learned about the health consequences of dietary behaviors linked to GI.

The second mini-program is the “Pregnancy Sugar Weight Record” mini-program. This mini-program offers 3 evidence-based modules: weight monitoring with personalized reference ranges based on prepregnancy BMI (pBMI), blood glucose monitoring with goals set with diabetes nurses, and an automated alert system for weight gain deviations. Weight measurements are standardized using calibrated digital scales and automatically synchronized with user profiles, with graphical trend visualization for weight gain deviations exceeding ±10% of target ranges.

The third mini-program is the “Fetal Weight Assessor” mini-program. This mini-program is designed for fetal growth monitoring, allowing participants to input key ultrasound parameters after routine prenatal examinations to estimate fetal weight. It also generates percentile curves to visualize growth trajectories relative to gestational age, aiding in comprehensive fetal growth assessment (Multimedia Appendix 1).

### Intervention Process

Upon enrollment, each participant received an individualized MNT prescription developed by a study dietitian using the “Diet Diary” mini-program, which incorporated personal parameters (age, gestational age, and pBMI) and specified precise gram weights of food types for daily meals and snacks. Participants recorded all food and beverage intake (type and weight) daily via the same mini-program, with digital food scales provided to support accurate measurement, and used the “Food Charts” self-learning module to learn as needed about food selection, GI values, and portion control techniques. Concurrently, women logged blood glucose and body weight data weekly into the “Pregnancy Sugar Weight Record” mini-program for continuous health monitoring. Those unable to input data independently could use paper records, audio or video recordings, or photos, with nurses assisting with data entry. Obstetricians delivered disease information to the participants, instructed them on the operation of the “Fetal Weight Assessor” mini-program, and supported the accurate input of ultrasound examination data during scheduled antenatal visits. Following this step, the system calculated and estimated fetal weight using the collected ultrasound parameters. A dynamic fetal weight trend curve was generated to visualize the changes in fetal weight over time.

**Figure 1.**
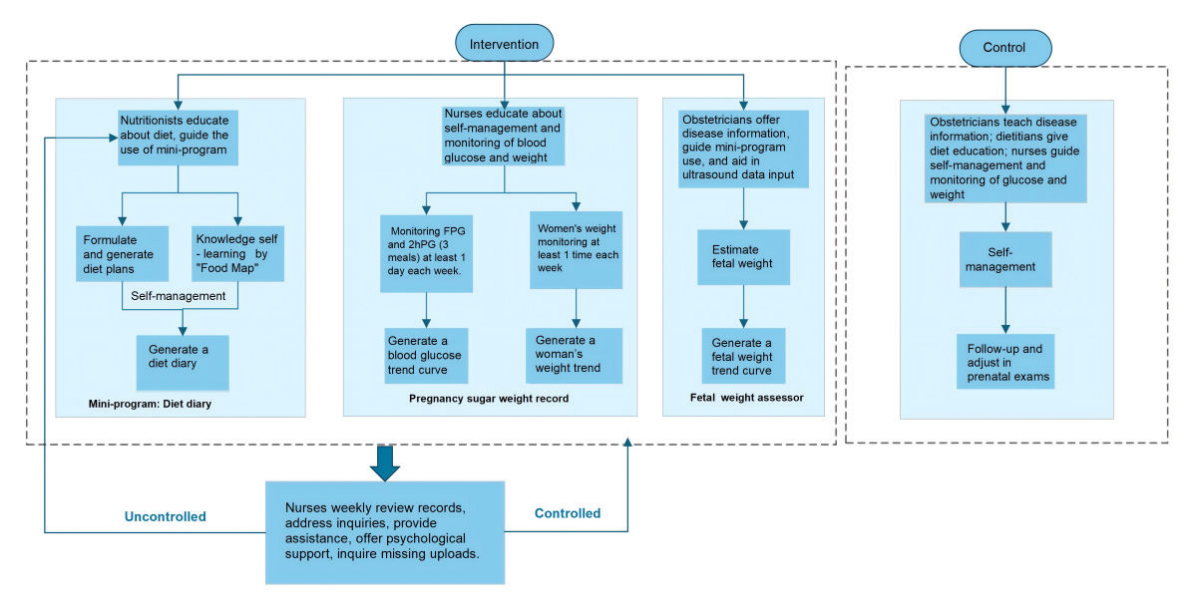
Implementation process of the study. FPG: fasting blood glucose; 2hPG: 2-hour postprandial glucose.

A multidisciplinary team (certified dietitians, nurses, and obstetricians) provided comprehensive support throughout the study. Diabetes nurses reviewed participants’ dietary logs, blood glucose levels, and weight records weekly via the WeChat backend, delivering personalized feedback and evaluations through WeChat messages or voice calls—acknowledging adherence achievements, addressing inquiries, and offering constructive suggestions. For participants with persistently unmet glycemic targets, nurses collaborated with dietitians to analyze issues and adjust meal plans (eg, modifying carbohydrate distribution or introducing alternative foods). A real-time consultation channel ensured that dietary inquiries received responses within 12 hours, and the team routinely audited 20% of the records to maintain intervention adherence standards. A nurse-modified WeChat peer support group facilitated weekly discussions in which women exchanged experiences, practical strategies, and insights into glycemic management and MNT implementation, fostering mutual encouragement. For participants facing persistent glucose management challenges for more than 2 weeks, the care team arranged 15 to 30 minutes of individual video consultations to provide focused support; these sessions could also lead to referrals for further multidisciplinary evaluation if needed. Individualized WeChat communications (messages, voice recordings, and video calls) were additionally provided for participants with specific needs, offering one-on-one dietary consultation and guidance. Throughout the intervention, all communications maintained an empathetic, nonjudgmental tone, and participants retained the flexibility to adjust their involvement or pause monitoring as needed. Figure 1 shows the implementation process of the study.

### Control Group: Standard Antenatal Care

Participants in the control group received routine GDM management in accordance with national guidelines. This included dietary guidance through printed educational materials, such as the Pagoda of Balanced Diet for Pregnant Women, which emphasized whole grains, lean proteins, and low-GI fruits. Biweekly 30-minute counseling sessions were conducted to address general dietary principles and portion sizes. Clinical monitoring involved regular weight, blood pressure, and capillary blood glucose measurements (fasting and postprandial) during scheduled antenatal visits, with participants recording self-monitored glucose values in paper logs. Additionally, exercise recommendations were provided to all study participants, including both the intervention and control groups, who were advised to engage in moderate-intensity walking for 30 minutes, 3 to 4 times weekly. Table 2 provides a detailed comparison of management approaches across modules between the WeMNT intervention and control groups.

**Table 2.** Comparison of management approaches across modules: WeChat-delivered medical nutrition therapy (WeMNT) versus standard care.

Component	Intervention group (WeMNT)	Control group
Dietary education, planning, and monitoring	Knowledge self-learning by “Food Charts” using the GI^a^ database (476 foods), AI^b^-driven personalized meal plans, and real-time nutrient monitoring and analysis	Static PDF guidelines and general portion advice
Weight monitoring	Automated IOM^c^ guideline alerts and trend visualization dashboards	Manual clinic measurements and verbal feedback
Glucose tracking	App-integrated data synchronization and weekly dietitian review	Paper-based logs and monthly physician review
Fetal surveillance	Ultrasound parameter input for fetal weight estimation and growth percentile curves	Standard ultrasound reports
Review, feedback, and support frequency	Daily self-monitoring+weekly professional feedback	Biweekly counseling+routine antenatal check-ups

a GI: glycemic index.

b AI: artificial intelligence.

c IOM: Institute of Medicine.

### Data Collection

Data collection was performed by research nurses who were blinded to group allocation. Baseline data were collected during the OGTT, which was conducted between the 24th and 28th gestational weeks, and the intervention was initiated at the 28th gestational week. Two intermediate follow-up assessments were planned from intervention initiation to delivery, scheduled at the 32nd (1-mo follow-up) and 36th weeks (2-mo follow-up). The study end point was defined as delivery, including preterm delivery (consistent with clinical criteria, ie, delivery between the 28th and 37th gestational weeks) and term delivery (delivery at or after the 38th gestational week). Participants with preterm delivery may miss the follow-up assessments scheduled thereafter and reach the study end point ahead of schedule (early closure) compared to term delivery (routine closure). Research nurses contacted participants by phone or WeChat to schedule data collection sessions, and all assessments were completed by participants in the obstetric clinic, with onsite researcher assistance available if needed. HbA_1c_ was measured twice, specifically at baseline and at the end point. To prevent contamination between groups, data collection procedures for the intervention and control groups were scheduled on separate dates at the study site.

### Ethical Considerations

This study was approved by the hospital institutional ethics committee (approval 2023-109-02) and abided by the principles of the Declaration of Helsinki. All patients who participated in the study participated voluntarily and obtained the informed consent from each patient in writing. Patients had the right to withdraw from the study at any time. All exported data must undergo anonymization by the data manager before statistical analysis can be conducted. Individuals cannot be identified in any results presentation. No financial compensation was provided to avoid coercion.

### Outcome Measures

The primary outcome measures of this study included glycemic control status and weight changes during pregnancy. Glycemic control was assessed using fasting blood glucose (FPG) and 2hPG levels, both of which were measured on a Hitachi 7600 analyzer (Hitachi High-Technologies Corporation) using venous plasma samples. Additionally, HbA_1c_ was determined through high-performance liquid chromatography. Gestational weight gain (GWG) was defined as the weight accrued during pregnancy and was computed as the numerical difference between the measured weight values at each follow-up time point and the baseline. The secondary outcome measures included obstetric complications and neonatal outcomes, such as gestational hypertension, preterm birth (<37 wk), and neonatal birth weight. The amniotic fluid index (AFI) was included as a secondary outcome because of its reported association with GDM-related fetal oligohydramnios and polyhydramnios, which may increase the risks of preterm labor and cesarean delivery. AFI was measured by ultrasound using the 4-quadrant technique after the 36th week and prior to delivery, with oligohydramnios defined as an AFI<5 cm and polyhydramnios as an AFI>25 cm, respectively. All measurements were conducted in strict adherence to standardized clinical practices.

### Statistical Analysis

**Figure 2.**
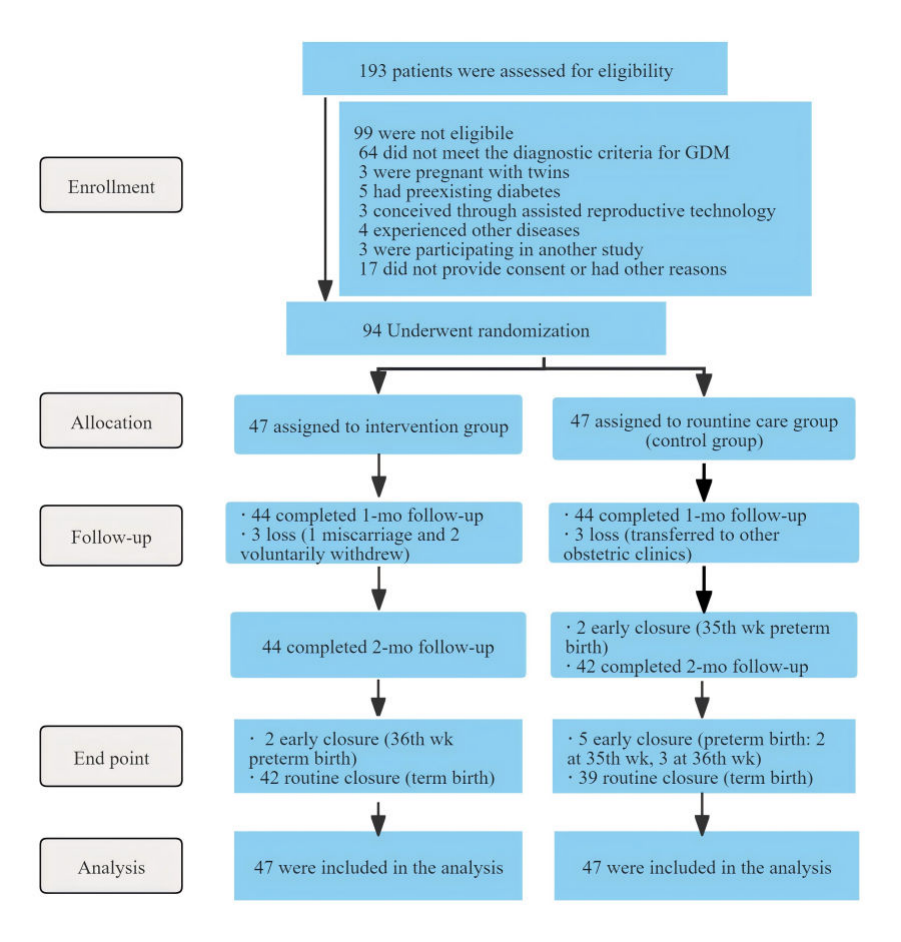
The participant flow chart of the study. GDM: gestational diabetes mellitus.

Descriptive statistical analyses were performed, whereby continuous variables were summarized as means (SDs) and categorical variables were presented as frequencies and corresponding percentages. The primary efficacy analysis adhered to the intention-to-treat (ITT) principle, wherein all randomized participants (n=94) were analyzed according to their original group assignments—this included individuals who were excluded during the study or lost to follow-up. Among the 94 participants, 6 participants (6.38%) had transferred to other clinics, experienced miscarriage, or withdrew (missing data, Figure 2). Little’s missing completely at random tests were performed on the postintervention outcome variables. The results were not statistically significant (*P*=.17 and *P>*.05), indicating that the missing data mechanism was completely at random.

Generalized linear mixed models (GLMMs) were used to assess the intervention’s effects on primary outcomes (FPG, 2hPG, and GWG) across different time points: 4 (time: baseline, 1 mo, 2 mo, and end point)×2 (group: intervention vs control). General sociodemographic characteristics and blood glucose management–related data were incorporated as covariates in this model to control for potential confounding factors. Additionally, a distinct 2 (time: baseline and end point)×2 (group: intervention vs control) GLMM was established to examine longitudinal changes in HbA_1c_ levels and to compare between-group differences in HbA_1c_. This analytical approach was selected because it can account for within-subject correlations and manage missing data under the missing-at-random assumption, enabling the use of all available data points without the need for multiple imputation. All GLMMs incorporated fixed effects for 3 key terms: the intervention group, time point, and the group×time interaction between group and time; a random intercept for each participant (1|ID) was also included to account for interindividual variability. To verify the robustness of the study findings, a per-protocol analysis was additionally performed, including only participants who completed the full intervention course and all scheduled follow-up assessments.

The secondary outcomes (maternal and fetal clinical measures) were analyzed using per-protocol methods. For the assessment of between-group disparities, we used independent-samples 2-tailed *t* tests to compare continuous variables (eg, AFI) and chi-square tests to analyze categorical variables such as the incidence of preeclampsia. All analyses were conducted using R software (R Foundation for Statistical Computing). The GLMMs were fitted using the lme4 package. A 2-sided *P* value <.05 was considered to indicate statistical significance.

## Results

### Overview

Between March 2023 and October 2023, a total of 94 participants were recruited for this study. Among them, 88 (93.62%) participants (mean age 32.18, SD 5.04 y) successfully completed the study: 44 (46.81%) participants in the intervention group and 44 (46.81%) in the control group. No adverse events or unintended effects were observed in either group throughout the study period. The participant flow and reasons for dropout are shown in Figure 2.

### Participant Engagement

The backend records of the WeChat app showed that in the intervention group, 87.2% (41/47) of the participants used the “Diet Diary” mini-program for daily dietary logging, whereas 78.7% (37/47) used the “Pregnancy Sugar Weight Record” and “Fetal Weight Assessor” programs for weight tracking. For the remaining participants who encountered difficulties inputting the data themselves, nurses assisted in entering the information into the mini-programs. All the participants explored the “food charts” module, learning the GI values of 476 foods, food selection, and weighing. Initially, they engaged with these tools 2 to 3 times daily for 20 to 45 minutes, but usage decreased as they became more familiar with food attributes. They only revisited the information when needed. Among the 44 participants who completed the study, 54.5% (24/44) adhered to the diet plan ≥80% of the days, 13.6% (6/44) followed it for 60% to 79% of the days, 11.4% (5/44) followed it for 40% to 59% of the days, another 11.4% (5/44) for 20% to 39% of the days, and 9.1% (4/44) for <20% of the days. In the control group, all 44 participants who completed the study reported independently studying the provided materials and following the dietary guidance for 60% to 80% of the days. No harms or unintended effects were found in each group.

### Baseline Demographic Characteristics and Clinical Data

The sample had a mean age of 32.18 (SD 5.04; range 22‐45) years (Table 3). The mean pBMI was 24.97 (SD 4) kg/m². For glycemic indicators, the mean FPG was 5.39 (SD 0.67) mmol/L, the mean 2hPG was 8.66 (SD 1.24) mmol/L, and the mean HbA_1c_ in the sample was 7.2% (SD 0.69).

**Table 3.** Baseline data across the intervention and control groups.

Characteristics	Total (N=94)	Intervention (n=47)	Control (n=47)
Age (y), mean (SD)	32.18 (5.04)	32.74 (4.91)	31.62 (5.15)
pBMI^a^ (kg/m^2^), mean (SD)	24.97 (4)	24.58 (4.47)	25.37 (3.48)
FPG^b^ (mmol/L), mean (SD)	5.39 (0.67)	5.37 (0.83)	5.40 (0.47)
2hPG^c^ (mmol/L), mean (SD)	8.66 (1.24)	8.70 (1.33)	8.41 (1.69)
HbA_1c_^d^ (%), mean (SD)	7.2 (0.69)	7.23 (0.68)	7.16 (0.71)
Weight status, n (%)			
Normal weight	48(51.0)	24 (51.06)	24 (51.06)
Overweight	37(39.4)	18 (38.30)	19 (40.43)
Obese	9 (9.6)	5 (10.64)	4 (8.51)
Household income (RMB per month), n (%)			
<3000	7 (7.4)	3 (6.38)	4 (8.51)
3000‐6000	24 (25.5)	20 (42.55)	17 (36.17)
6000‐9000	40 (42.6)	19 (40.43)	20 (42.55)
>9000	23 (24.5)	5 (10.64)	6 (12.77)
Education years, n (%)			
1‐9	8 (8.5)	6 (12.77)	2 (4.26)
10‐12	30 (31.9)	14 (29.79)	16 (34.04)
13‐16	45 (47.9)	21 (44.68)	24 (51.06)
≥17	11(11.7)	6 (12.77)	5 (10.64)

a pBMI: prepregnancy BMI.

b FPG: fasting blood glucose.

c 2hPG: 2-hour postprandial glucose.

d HbA_1c_: hemoglobin A_1c_.

### Changes in Primary Outcomes Over Time Between the mHealth and Control Groups

**Figure 3.**
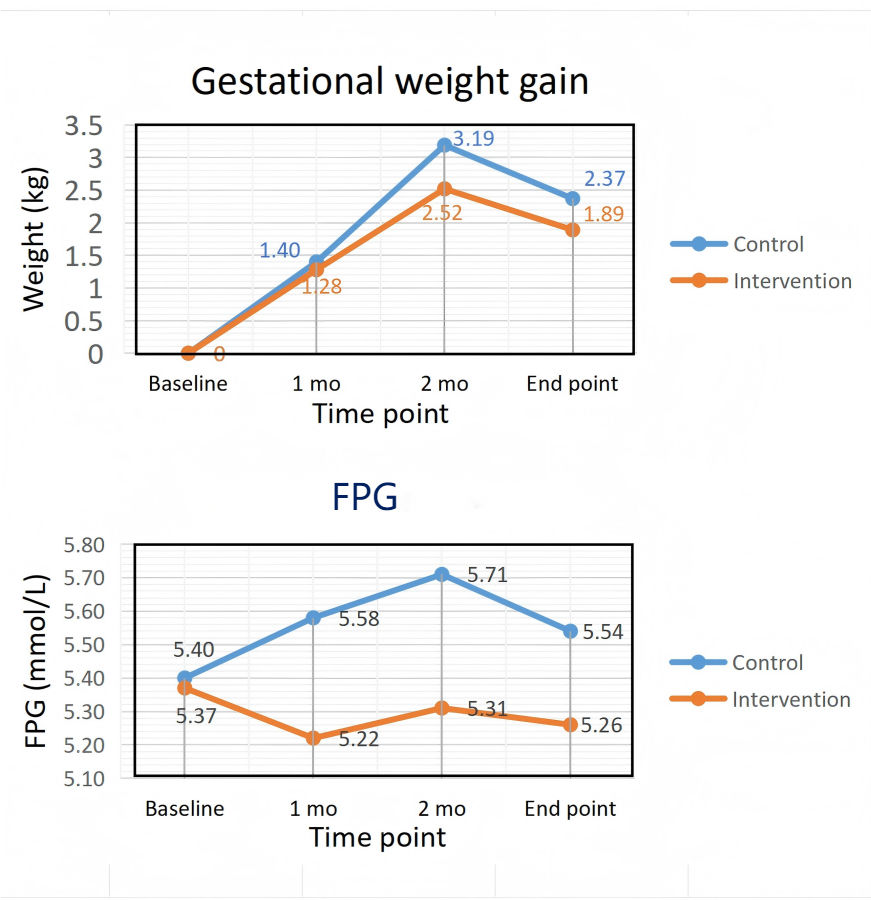
Changes in gestational weight gain and fasting blood glucose (FPG) between the intervention and control groups over time.

The GWG progressively increased over time in both groups, with the control group exhibiting greater increases (Figure 3). By the end point, the control group’s mean weight had increased by 15.11 (SD 12.56) kg, whereas the intervention group’s mean weight had increased by 13.31 (SD 12.21) kg (Table 4).

FPG levels fluctuated between the groups (Figure 3). In the control group, the FPG concentration increased by 0.17 (SD 0.61) mmol/L at 1 month and 0.32 (SD 0.70) mmol/L at 2 months but then slightly decreased by 0.15 (SD 0.62) mmol/L at the end point (Table 4). In contrast, the levels in the intervention group gradually decreased, with FPG levels decreasing by 0.15 (SD 0.70) mmol/L at 1 month, 0.06 (SD 0.78) mmol/L at 2 months, and 0.11 (SD 0.56) mmol/L at the end point.

**Table 4.** Comparisons of the primary outcomes between the intervention and control groups.

Variables	Control group				Intervention group			
	Baseline (n=47)	1 mo (n=44)	2 mo (n=44)	End point (n=44)	Baseline (n=47)	1 mo (n=44)	2 mo (n=42)	End point (n=44)
GWG^a^ (kg), mean (SD)	0	1.40 (0.37)	3.19 (0.77)	2.37 (0.67)	0	1.18 (0.32)	2.52 (0.63)	1.89 (0.51)
FPG^b^ (mmol/L), mean (SD)	5.39 (0.49)	5.56 (0.61)	5.71 (0.70)	5.54 (0.62)	5.37 (0.86)	5.22 (0.70)	5.31 (0.78)	5.26 (0.56)
2hPG^c^ (mmol/L), mean (SD)	8.49 (1.08)	8.62 (1.38)	8.79 (1.21)	8.91 (1.23)	8.60 (1.30)	8.34 (1.69)	8.25 (1.56)	8.08 (1.51)
HbA_1c_^d^^,^^e^ (%), mean (SD)	7.14 (0.72)	N/A^f^	N/A	6.71 (0.62)	7.21 (0.70)	N/A	N/A	6.29 (0.75)

a GWG: gestational weight gain

b FPG: fasting blood glucose.

c 2hPG: 2-hour postprandial glucose.

d HbA_1c_: hemoglobin A_1c_.

e HbA_1c_ was measured only at baseline and at the final end point of the study.

f N/A: not applicable.

**Figure 4.**
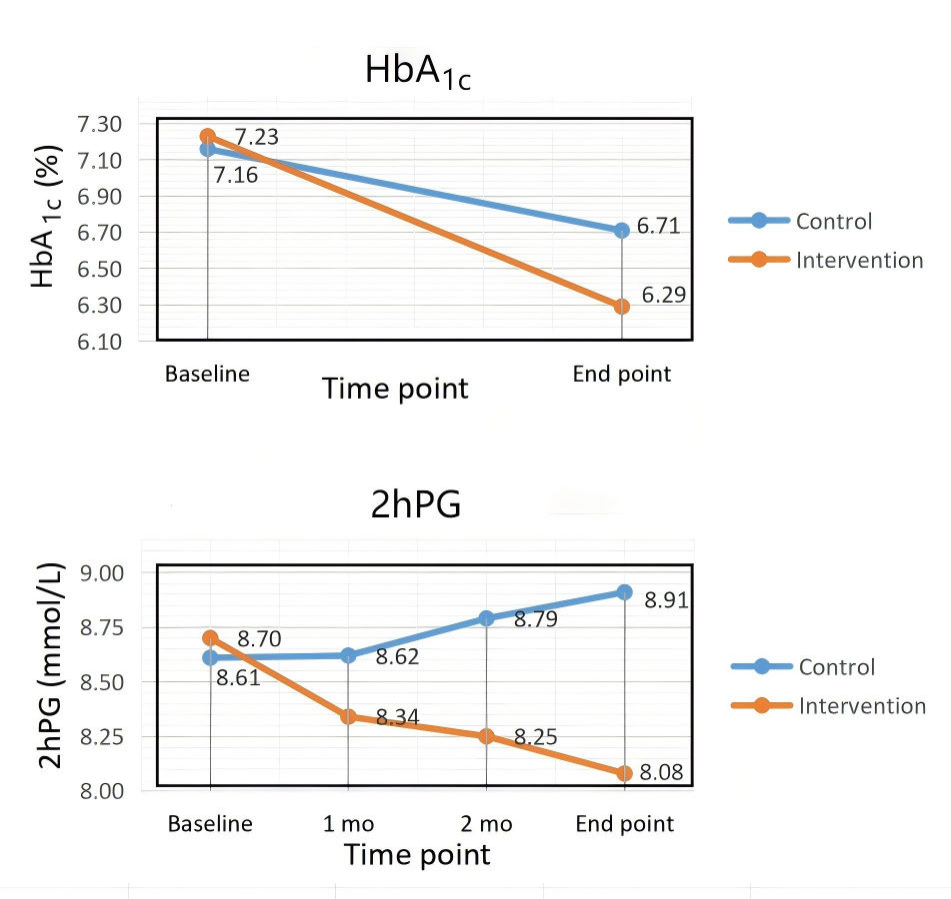
Changes in hemoglobin A1c (HbA_1c_) and 2-hour postprandial glucose (2hPG) between the intervention and control groups over time.

Both groups showed a decreasing trend in HbA_1c_ levels (Figure 4), with the intervention group demonstrating substantially greater decreases. By the end point, the control group’s mean HbA_1c_ had decreased to 6.71% (SD 0.62), representing a reduction of 0.43% (Table 4). The mean HbA_1c_ in the intervention group decreased to 6.29% (SD 0.75), indicating a more pronounced reduction of 0.92% from baseline.

Both groups showed an increase in 2hPG over time, but the intervention group demonstrated greater decreases (Figure 4). By the end point, the control group’s mean 2hPG had risen to 8.91 (SD 1.23) mmol/L, representing an increase of 0.42 mmol/L (Table 4). In contrast, the mean 2hPG of the intervention group decreased to 8.08 (SD 1.51) mmol/L, indicating a reduction of 0.52 mmol/L from baseline.

### Effectiveness of the Intervention Through the ITT Analysis

Adjusted GLMMs with ITT analysis revealed a significant group×time interaction for GWG (Table 5); notably, the intervention group achieved a significantly greater reduction in GWG over time compared with the control group, as indicated by the negative coefficient of the group×time interaction (β=−1.96, 95% CI −3.58 to −0.34; *P*=.02). For FPG, no significant group×time interaction effect was observed. In contrast, both 2hPG and HbA_1c_ showed significant group×time interaction effects. Compared with the control group, the intervention group demonstrated substantially greater decreases in 2hPG (β=−0.12, 95% CI −0.19 to −0.04; *P*<.001) and HbA_1c_ (β=−0.49, 95% CI −0.74 to −0.23; *P*<.001) over time. The results of the per-protocol analyses (Multimedia Appendix 2) were consistent in terms of the direction and significance of the ITT analyses.

**Table 5.** Generalized linear mixed model analyses of the efficacy of the intervention on the primary outcomes.

Outcome	β estimate (95% CI)	SE	*P* value
GWG^a^ (kg)			
Time	15.10 (13.96 to 16.25)	0.59	<.001
Group	−2.29 (−7.24 to 2.67)	2.53	.37
Group×time	−1.96 (−3.58 to −0.34)	0.83	.02
FPG^b^ (mmol/L)			
Time	0.02 (−0.02 to 0.07)	0.02	.26
Group	−0.01 (−0.06 to 0.04)	0.02	.65
Group×time	−0.04 (−0.10 to 0.02)	0.03	.18
2hPG^c^ (mmol/L)			
Time	0.04 (−0.02 to 0.09)	0.03	.22
Group	0.02 (−0.05 to 0.08)	0.03	.65
Group×time	−0.12 (−0.19 to −0.04)	0.04	<.001
HbA_1c_^d^ (%)			
Time	−0.44 (−0.62 to −0.26)	0.09	<.001
Group	0.07 (−0.21 to 0.35)	0.14	.64
Group×time	−0.49 (−0.74 to −0.23)	0.13	<.001

a GWG: gestational weight gain.

b FPG: fasting blood glucose.

c 2hPG: 2-hour postprandial glucose.

d HbA_1c_: hemoglobin A_1c_.

### Maternal and Fetal Outcomes

No unexpected maternal events were observed among the participants. Compared with that in the control group, the AFI in the intervention group was significantly different (Table 6). However, both values were within the normal range and were not expected to have adverse effects on the fetuses. The number of cases of preeclampsia, preterm infants, low-birth-weight infants (birth weight <2500 g), and macrosomia (birth weight >4000 g) were lower in the intervention group than those in the control group, yet the differences were not statistically significant (Table 6).

**Table 6.** Comparisons of the secondary outcomes between the intervention and control groups.

Group	AFI^a^^,^^b^^,^^c^ (cm), mean (SD)	Preeclampsia^d^^,^^e^, n (%)	Preterm birth^f^^,^^g^, n (%)	Low birth weight^h^^,^^i^, n (%)	Macrosomia^j^^,^^k^, n (%)
Intervention (n=44)	12.01 (3.31)	1 (2.27)	2 (4.54)	1 (2.27)	4 (9.09)
Control (n=44)	13.49 (3.19)	3 (6.82)	5 (11.36)	3 (6.82)	7 (15.91)

a AFI: amniotic fluid index.

b *t*_86_=−2.15 (2 sided).

c *P*=.04.

d *Χ*2_1_=0.26.

e *P*=.60.

f *Χ*2_1_=0.62.

g *P*=.43.

h *Χ*2_1_=0.26.

i *P*=.60.

j *Χ*2_1_=0.94.

k *P*=.33.

## Discussion

### Principal Findings

Collectively, the WeMNT intervention demonstrated targeted efficacy in improving glycemic control among women with GDM, particularly in modulating GWG, 2hPG, and HbA_1c_ dynamics. While it failed to have a meaningful effect on FPG, the favorable changes in 2hPG and HbA_1c_ trajectories highlight the intervention’s potential as a practical tool for GDM management. These findings underscore the importance of tailored dietary interventions to address specific glycemic outcomes during pregnancy, with further research needed to optimize the interventions for broader efficacy.

Prior studies on HbA_1c_ management have demonstrated that WeChat-delivered intervention tools—validated across 6 RCTs—yield a notable effect size of 0.84 (95% CI –0.22 to −1.46) [21]. Remarkably, in our MNT intervention cohort, HbA_1c_ levels substantially decreased. This promising outcome is most likely due to the mHealth technology we crafted. An mHealth intervention based on a behavioral model, whether used independently or in tandem with other approaches, confers a distinct advantage for effective blood glucose control [25]. Continuous self-tracking of blood glucose, a common intervention measure in GDM management, clarifies the links between glucose levels and nutrition, thereby supporting autonomous self-management without the need for direct health care assistance [26]. Using key BCTs in diabetes management, our project provides timely feedback and evaluations based on records and indicator data, acknowledging strengths and making improvement suggestions through WeChat instant messages and voice calls. It not only facilitates self-learning and monitoring of GDM and communication with health care professionals but also establishes WeChat peer groups to encourage experience sharing and mutual encouragement among patients. This feature effectively sustains participants’ engagement and active involvement in managing their own care, a critical aspect of the intervention [27,28].

Evidence indicates that when HbA_1c_ levels are less than 7.3%, 2hPG may contribute up to 70% of total HbA_1c_ elevation [29]. In our GDM cohort, FPG had a mean of 5.38 (SD 0.69) mmol/L, with an average HbA_1c_ of 7.17% (SD 0.71%), suggesting that the increase in HbA_1c_ in this population is largely attributable to increased 2hPG levels. Given this relationship, dietary variations have a pronounced effect on 2hPG, rendering this parameter highly responsive to dietary interventions; HbA_1c_ consequently shares this sensitivity, as its elevation is closely linked to 2hPG fluctuations. In contrast, the intervention yielded no significant effects on FPG levels. These findings are consistent with the results of a meta-analysis focusing on dietary interventions for GDM, which reported a significant 2hPG reduction of 27.78 mg/dL (*P*<.001) and a transient FPG reduction of 24.07 mg/dL (*P*=.02) in intervention groups [6]; however, only the abovementioned results align with common observations in the field: FPG levels often do not significantly respond to dietary interventions in individuals with GDM [30].

Maternal weight gain throughout pregnancy constitutes a fundamental physiological process of gestation. Our regression model revealed a highly significant time main effect (β=15.10, 95% CI 13.96‐16.25; *P*<.001), indicating that a substantial increase in maternal weight occurs as pregnancy progresses. Notably, mobile phones have emerged as a promising tool for GWG management in recent years. Prior studies have consistently demonstrated that smartphone apps exhibit remarkable efficacy in promoting healthy weight gain during pregnancy [31,32]. These technologies overcome the limitations of traditional face-to-face guidance by providing personalized dietary recommendations, supporting pregnant women in maintaining optimal weight trajectories during gestation. The findings of this study validated the effectiveness of mHealth interventions in controlling GWG. Our regression analysis revealed a significant group×time interaction effect (β=−1.96, 95% CI −3.58 to −0.34; *P*=.02), indicating a significantly gentler pattern of weight gain in the intervention group compared with the control group. While the effect size was small and its impact on final weight gain was limited, its value in controlling dynamic weight growth remained undiminished. These results align with the conclusions of previous studies, further confirming the potential of mHealth tools as an adjunct to routine antenatal care.

The unique advantages of our mHealth intervention lie in its targeted timing and strategy design, which address the unmet needs of pregnant women in the third trimester. Our intervention started at week 28 (the start of the third trimester), a time when physical constraints hinder exercise-based weight management [25]. Unlike exercise-reliant programs, it integrates core nutritional guidance (BCT 4.1/4.2), MNT plan design support (BCT 6.1), scheduled meal reminders (BCT 1.4), food portion logging, and real-time intake target alerts—helping participants use science-backed diets to curb excessive weight gain amid inevitable late-pregnancy physiological weight growth. A second key advantage is its focus on collaborative clinical and self-management efforts, which are critical for addressing inappropriate GWG and protecting maternal and child health. This dual-focused approach bridges the gap between clinical recommendations and real-world adherence.

Regarding other maternal and fetal outcomes, a comprehensive meta-analysis of 32 RCTs confirmed that mHealth strategies effectively improve glycemic control and reduce the risks of adverse maternal and fetal outcomes [19]. Specifically, the risk ratios were 0.49 for macrosomia (95% CI 0.30‐0.80; *P*<.01), 0.48 for preeclampsia (95% CI 0.40‐0.58; *P*<.01), and 0.27 for preterm birth (95% CI 0.20‐0.35; *P*<.01). However, our study revealed no significant effects of the intervention on these outcomes or birth weight. Notably, our limited sample size may have restricted the detection of subtle effects, highlighting the need for further investigation of these endpoints in future research.

### Limitations

This study has several limitations. First, it was impossible to implement blinding for the personnel involved in the WeMNT intervention. To address this issue, we crafted a protocol to guide the intervention, refined the guidance to align with it, and equipped the team with the skills to execute it effectively. Second, this study, which was conducted in a single region with a homogeneous population, has limitations in terms of generalizability. To bridge the implementation gap, we documented each mHealth module’s design and implementation, aligned with those of the BCW. This supports cross-setting validation and real-world generalization. Third, our study focused on short-term outcomes, and long-term effects on mothers and children remain undetermined. Future research should recruit larger, heterogeneous cohorts to evaluate the efficacy across diverse populations and extended timeframes. Finally, although we adjusted for key covariates in the sensitivity analyses, there may still be unmeasured confounders (eg, genetic predisposition and family medical history) that could influence the study outcomes. Future research should use multivariate regression or mixed effects models to achieve more effective control over these factors.

### Conclusions

This study demonstrated that the WeMNT program effectively improved nutritional self-management and blood glucose control in patients, with significant reductions in 2hPG, HbA_1c_, and GWGs, supporting its clinical adoption for GDM. However, long-term maternal-child outcomes remain unexplored and warrant larger, diverse cohort studies for extended evaluation.

## Supplementary material

10.2196/67410 Multimedia Appendix 1 Screenshots of the WeChat mini-programs in the WeMNT intervention.

10.2196/67410 Multimedia Appendix 2 The results of the per-protocol analyses of the efficacy of the intervention on the primary outcome.

10.2196/67410 Checklist 1 CONSORT-EHEALTH (V 1.6.1) checklist.

## Abbreviations

2hPG: 2-hour postprandial glucose
AFI: amniotic fluid index
BCT: behavior change technique
BCW: behavior change wheel
COM-B: capability, opportunity, motivation, and behavior
CONSORT: Consolidated Standards of Reporting Trials
FPG: fasting blood glucose
GDM: gestational diabetes mellitus
GI: glycemic index
GLMM: generalized linear mixed model
GWG: gestational weight gain
HbA_1c_: hemoglobin A_1c_
ITT: intention-to-treat
mHealth: mobile health
MNT: medical nutrition therapy
OGTT: oral glucose tolerance test
pBMI: prepregnancy BMI
RCT: randomized controlled trial
WeMNT: WeChat-delivered medical nutrition therapy
