# Supplementary material for: WeChat-Delivered Mobile Medical Nutrition Therapy Intervention in Gestational Diabetes Mellitus: Randomized Controlled Trial
Source: JMIR Mhealth Uhealth. 2026 Jun 11;14:e67410. doi: 10.2196/67410 (PMC13257780; doi:10.2196/67410)
Supplement: Multimedia Appendix 1 [file mhealth-v14-e67410-s001.pdf]

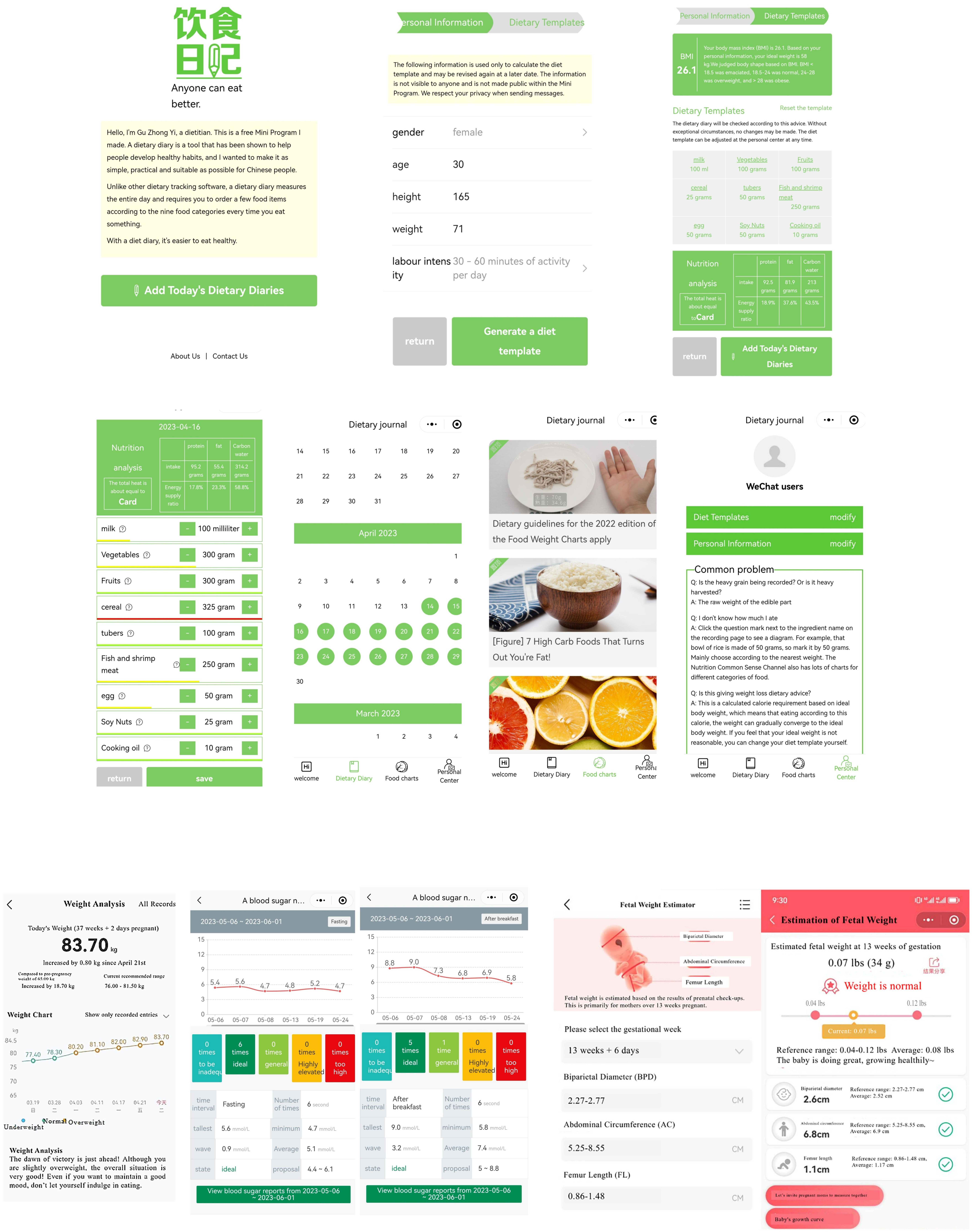
饮食日记
Anyone can eat better.
Hello, I'm Gu Zhong Yi, a dietitian. This is a free Mini Program I made. A dietary diary is a tool that has been shown to help people develop healthy habits, and I wanted to make it as simple, practical and suitable as possible for Chinese people.
Unlike other dietary tracking software, a dietary diary measures the entire day and requires you to order a few food items according to the nine food categories every time you eat something.
With a diet diary, it's easier to eat healthy.
Add Today's Dietary Diaries
About Us | Contact Us
ersonal Information
Dietary Templates
The following information is used only to calculate the diet template and may be revised again at a later date. The information is not visible to anyone and is not made public within the Mini Program. We respect your privacy when sending messages.
gender female
age 30
height 165
weight 71
labour intensity 30 - 60 minutes of activity per day
return
Generate a diet template
Personal Information
Dietary Templates
BMI 26.1
Your body mass index (BMI) is 26.1. Based on your personal information, your ideal weight is 58 kg.We judged body shape based on BMI. BMI < 18.5 was emaciated, 18.5-24 was normal, 24-28 was overweight, and > 28 was obese.
Dietary Templates
Reset the template
The dietary diary will be checked according to this advice. Without exceptional circumstances, no changes may be made. The diet template can be adjusted at the personal center at any time.
milk 100 ml
Vegetables 100 grams
Fruits 100 grams
cereal 25 grams
tubers 50 grams
Fish and shrimp meat 250 grams
egg 50 grams
Soy Nuts 50 grams
Cooking oil 10 grams
Nutrition analysis
protein fat Carbon water
intake 92.5 grams 81.9 grams 213 grams
Energy supply ratio 18.9% 37.6% 43.5%
The total heat is about equal to Card
return
Add Today's Dietary Diaries
2023-04-16
Nutrition analysis
protein fat Carbon water
intake 95.2 grams 55.4 grams 314.2 grams
Energy supply ratio 17.8% 23.3% 58.8%
The total heat is about equal to Card
milk 100 milliliter
Vegetables 300 gram
Fruits 300 gram
cereal 325 gram
tubers 100 gram
Fish and shrimp meat 250 gram
egg 50 gram
Soy Nuts 25 gram
Cooking oil 10 gram
return
save
Dietary journal
14 15 16 17 18 19 20
21 22 23 24 25 26 27
28 29 30 31
April 2023
1
2 3 4 5 6 7 8
9 10 11 12 13 14 15
16 17 18 19 20 21 22
23 24 25 26 27 28 29
30
March 2023
1 2 3 4
welcome
Dietary Diary
Food charts
Personal Center
Dietary journal
Dietary guidelines for the 2022 edition of the Food Weight Charts apply
[Figure] 7 High Carb Foods That Turns Out You're Fat!
welcome
Dietary Diary
Food charts
Personal Center
Dietary journal
WeChat users
Diet Templates modify
Personal Information modify
Common problem
Q: Is the heavy grain being recorded? Or is it heavy harvested?
A: The raw weight of the edible part
Q: I don't know how much I ate
A: Click the question mark next to the ingredient name on the recording page to see a diagram. For example, that bowl of rice is made of 50 grams, so mark it by 50 grams. Mainly choose according to the nearest weight. The Nutrition Common Sense Channel also has lots of charts for different categories of food.
Q: Is this giving weight loss dietary advice?
A: This is a calculated calorie requirement based on ideal body weight, which means that eating according to this calorie, the weight can gradually converge to the ideal body weight. If you feel that your ideal weight is not reasonable, you can change your diet template yourself.
welcome
Dietary Diary
Food charts
Personal Center
Weight Analysis
All Records
Today's Weight (37 weeks + 2 days pregnant)
83.70 kg
Increased by 0.80 kg since April 21st
Compared to pre-pregnancy weight of 65.00 kg
Increased by 18.70 kg
Current recommended range
76.00 - 81.50 kg
Weight Chart
Show only recorded entries
kg
84.5 80 75 70 65
77.40 78.30 80.20 81.10 82.00 82.90 83.70
03.19 03.28 04.03 04.11 04.17 04.21 今天
Underweight
Normal
Overweight
Weight Analysis
The dawn of victory is just ahead! Although you are slightly overweight, the overall situation is very good! Even if you want to maintain a good mood, don't let yourself indulge in eating.
A blood sugar n...
2023-05-06 ~ 2023-06-01
Fasting
15 12 9 6 3 0
5.4 5.6 4.7 4.8 5.2 4.7
05-06 05-07 05-08 05-13 05-19 05-24
0 times to be inadequ
6 times ideal
0 times general
0 times Highly elevated
0 times too high
time interval Fasting
Number of times 6 second
tallest 5.6 mmol/L
minimum 4.7 mmol/L
wave 0.9 mmol/L
Average 5.1 mmol/L
state ideal
proposal 4.4 ~ 6.1
View blood sugar reports from 2023-05-06 ~ 2023-06-01
A blood sugar n...
2023-05-06 ~ 2023-06-01
After breakfast
15 12 9 6 3 0
8.8 9.0 7.3 6.8 6.9 5.8
05-06 05-07 05-08 05-13 05-19 05-24
0 times to be inadequ
5 times ideal
1 time general
0 times Highly elevated
0 times too high
time interval After breakfast
Number of times 6 second
tallest 9.0 mmol/L
minimum 5.8 mmol/L
wave 3.2 mmol/L
Average 7.4 mmol/L
state ideal
proposal 5 ~ 8.8
View blood sugar reports from 2023-05-06 ~ 2023-06-01
Fetal Weight Estimator
Biparietal Diameter
Abdominal Circumference
Femur Length
Fetal weight is estimated based on the results of prenatal check-ups. This is primarily for mothers over 13 weeks pregnant.
Please select the gestational week
13 weeks + 6 days
Biparietal Diameter (BPD)
2.27-2.77 CM
Abdominal Circumference (AC)
5.25-8.55 CM
Femur Length (FL)
0.86-1.48 CM
9:30
Estimation of Fetal Weight
Estimated fetal weight at 13 weeks of gestation
0.07 lbs (34 g)
结果分享
Weight is normal
0.04 lbs
0.12 lbs
Current: 0.07 lbs
Reference range: 0.04-0.12 lbs Average: 0.08 lbs
The baby is doing great, growing healthily~
Biparietal diameter 2.6cm Reference range: 2.27-2.77 cm Average: 2.52 cm
Abdominal circumference 6.8cm Reference range: 5.25-8.55 cm, Average: 6.9 cm
Femur length 1.1cm Reference range: 0.86-1.48 cm, Average: 1.17 cm
Let's invite pregnant moms to measure together
Baby's growth curve
