# Supplementary material for: WeChat-Delivered Mobile Medical Nutrition Therapy Intervention in Gestational Diabetes Mellitus: Randomized Controlled Trial
Source: JMIR Mhealth Uhealth. 2026 Jun 11;14:e67410. doi: 10.2196/67410 (PMC13257780; doi:10.2196/67410)
Supplement: Multimedia Appendix 2 [file mhealth-v14-e67410-s002.docx]

Multimedia Appendix 1.

The results of the per protocol analyses of the efficacy of the intervention on the primary outcome.

| Outcome | | β estimate | SE | 95%CI | *P* value |
| --- | --- | --- | --- | --- | --- |
| GWG (kg) | |  |  |  |  |
|  | Time | 15.11 | 0.58 | 13.97 to 16.25 | <.001 |
|  | Group | -2.96 | 2.48 | -7.82 to 1.89 | .24 |
|  | Group×time | -1.80 | 0.82 | -3.40 to -0.19 | .03 |
| FPG (mmol/L) | |  |  |  |  |
|  | Time | 0.03 | 0.02 | -0.02 to 0.07 | .25 |
|  | Group | -0.01 | 0.03 | -0.06 to 0.04 | .72 |
|  | Group×time | -0.04 | 0.03 | -0.10 to 0.02 | .17 |
| 2hPG (mmol/L) | |  |  |  |  |
|  | Time | 0.04 | 0.03 | -0.02 to 0.09 | .12 |
|  | Group | 0.02 | 0.03 | -0.05 to 0.08 | .61 |
|  | Group×time | -0.12 | 0.04 | -0.19 to -0.04 | .004 |
| HbA1c (%) | |  |  |  |  |
|  | Time | -0.42 | 0.09 | -0.62 to -0.24 | <.001 |
|  | Group | 0.07 | 0.15 | -0.22 to 0.37 | .62 |
|  | Group×time | -0.48 | 0.13 | -0.75 to -0.23 | <.001 |
