# Supplementary material for: WeChat-Delivered Mobile Medical Nutrition Therapy Intervention in Gestational Diabetes Mellitus: Randomized Controlled Trial
Source: JMIR Mhealth Uhealth. 2026 Jun 11;14:e67410. doi: 10.2196/67410 (PMC13257780; doi:10.2196/67410)
Supplement: Checklist 1 [file mhealth-v14-e67410-s003.pdf]

CONSORT-EHEALTH (V 1.6.1) - Submission/Publication Form

The CONSORT-EHEALTH checklist is intended for authors of randomized trials evaluating web-based and Internet-based applications/interventions, including mobile interventions, electronic games (incl multiplayer games), social media, certain telehealth applications, and other interactive and/or networked electronic applications. Some of the items (e.g. all subitems under item 5 - description of the intervention) may also be applicable for other study designs.

The goal of the CONSORT EHEALTH checklist and guideline is to be
a) a guide for reporting for authors of RCTs,
b) to form a basis for appraisal of an ehealth trial (in terms of validity)

CONSORT-EHEALTH items/subitems are MANDATORY reporting items for studies published in the Journal of Medical Internet Research and other journals / scientific societies endorsing the checklist.

Items numbered 1., 2., 3., 4a., 4b etc are original CONSORT or CONSORT-NPT (non-pharmacologic treatment) items.
Items with Roman numerals (i., ii, iii, iv etc.) are CONSORT-EHEALTH extensions/clarifications.

As the CONSORT-EHEALTH checklist is still considered in a formative stage, we would ask that you also RATE ON A SCALE OF 1-5 how important/useful you feel each item is FOR THE PURPOSE OF THE CHECKLIST and reporting guideline (optional).

Mandatory reporting items are marked with a red *.
In the textboxes, either copy & paste the relevant sections from your manuscript into this form - please include any quotes from your manuscript in QUOTATION MARKS,
or answer directly by providing additional information not in the manuscript, or elaborating on why the item was not relevant for this study.

YOUR ANSWERS WILL BE PUBLISHED AS A SUPPLEMENTARY FILE TO YOUR PUBLICATION IN JMIR AND ARE CONSIDERED PART OF YOUR PUBLICATION (IF ACCEPTED).
Please fill in these questions diligently. Information will not be copyedited, so please use proper spelling and grammar, use correct capitalization, and avoid abbreviations.

DO NOT FORGET TO SAVE AS PDF _AND_ CLICK THE SUBMIT BUTTON SO YOUR ANSWERS ARE IN OUR DATABASE !!!

Citation Suggestion (if you append the pdf as Appendix we suggest to cite this paper in the caption):
Eysenbach G, CONSORT-EHEALTH Group
CONSORT-EHEALTH: Improving and Standardizing Evaluation Reports of Web-based and Mobile Health Interventions
J Med Internet Res 2011;13(4):e126

URL: http://www.jmir.org/2011/4/e126/
doi: 10.2196/jmir.1923
PMID: 22209829

登录 Google 即可保存进度。了解详情

* 表示必填

Your name *

First Last

Lining Mu

Primary Affiliation (short), City, Country *

University of Toronto, Toronto, Canada

Shandong First Medical University,

Jinan Central Hospital, Jinan, China

Your e-mail address *

abc@gmail.com

mlnsubmi@163.com

Title of your manuscript *

Provide the (draft) title of your manuscript.

WeChat-Delivered Mobile Medical Nutrition Therapy Intervention in Gestational Diabetes Mellitus: A Randomized Controlled Trial

Name of your App/Software/Intervention *

If there is a short and a long/alternate name, write the short name first and add the long name in brackets.

WeMNT, a WeChat-delivered Medical nutrition [illegible]

Evaluated Version (if any)

e.g. "V1", "Release 2017-03-01", "Version 2.0.27913"

revised version 20260115

Language(s) *

What language is the intervention/app in? If multiple languages are available, separate by comma (e.g. "English, French")

English, Chinese, Spanish, Korean, Japanese, F[illegible]

URL of your Intervention Website or App

e.g. a direct link to the mobile app on app in appstore (itunes, Google Play), or URL of the website. If the intervention is a DVD or hardware, you can also link to an Amazon page.

您的回答

URL of an image/screenshot (optional)

您的回答

Accessibility *

Can an enduser access the intervention presently?

- (●) access is free and open
- ( ) access only for special usergroups, not open
- ( ) access is open to everyone, but requires payment/subscription/in-app purchases
- ( ) app/intervention no longer accessible
- ( ) 其他:

Primary Medical Indication/Disease/Condition *

e.g. "Stress", "Diabetes", or define the target group in brackets after the condition, e.g. "Autism (Parents of children with)", "Alzheimers (Informal Caregivers of)"

Gestational diabetes mellitus (GDM)

Primary Outcomes measured in trial *

comma-separated list of primary outcomes reported in the trial

fasting blood glucose (FPG), 2-hour post-pran

Secondary/other outcomes

Are there any other outcomes the intervention is expected to affect?

obstetric complications and neonatal parameters

Recommended "Dose" *

What do the instructions for users say on how often the app should be used?

- (●) Approximately Daily
- ( ) Approximately Weekly
- ( ) Approximately Monthly
- ( ) Approximately Yearly
- ( ) "as needed"
- ( ) 其他:

Approx. Percentage of Users (starters) still using the app as recommended after 3 months *

- ( ) unknown / not evaluated
- ( ) 0-10%
- ( ) 11-20%
- ( ) 21-30%
- ( ) 31-40%
- ( ) 41-50%
- ( ) 51-60%
- ( ) 61-70%
- ( ) 71%-80%
- ( ) 81-90%
- (●) 91-100%
- ( ) 其他:

Overall, was the app/intervention effective? *

- ( ) yes: all primary outcomes were significantly better in intervention group vs control
- (x) partly: SOME primary outcomes were significantly better in intervention group vs control
- ( ) no statistically significant difference between control and intervention
- ( ) potentially harmful: control was significantly better than intervention in one or more outcomes
- ( ) inconclusive: more research is needed
- ( ) 其他:

Article Preparation Status/Stage *

At which stage in your article preparation are you currently (at the time you fill in this form)

- ( ) not submitted yet - in early draft status
- ( ) not submitted yet - in late draft status, just before submission
- ( ) submitted to a journal but not reviewed yet
- (x) submitted to a journal and after receiving initial reviewer comments
- ( ) submitted to a journal and accepted, but not published yet
- ( ) published
- ( ) 其他:

Journal *

If you already know where you will submit this paper (or if it is already submitted), please provide the journal name (if it is not JMIR, provide the journal name under "other")

- ○ not submitted yet / unclear where I will submit this
- ○ Journal of Medical Internet Research (JMIR)
- ◉ JMIR mHealth and UHealth
- ○ JMIR Serious Games
- ○ JMIR Mental Health
- ○ JMIR Public Health
- ○ JMIR Formative Research
- ○ Other JMIR sister journal
- ○ 其他:

Is this a full powered effectiveness trial or a pilot/feasibility trial? *

- ○ Pilot/feasibility
- ◉ Fully powered

Manuscript tracking number *

If this is a JMIR submission, please provide the manuscript tracking number under "other" (The ms tracking number can be found in the submission acknowledgement email, or when you login as author in JMIR. If the paper is already published in JMIR, then the ms tracking number is the four-digit number at the end of the DOI, to be found at the bottom of each published article in JMIR)

- ○ no ms number (yet) / not (yet) submitted to / published in JMIR
- ◉ 其他: #67410

TITLE AND ABSTRACT

1a) TITLE: Identification as a randomized trial in the title

1a) Does your paper address CONSORT item 1a? *

I.e does the title contain the phrase "Randomized Controlled Trial"? (if not, explain the reason under "other")

- (●) yes
- ( ) 其他:

1a-i) Identify the mode of delivery in the title

Identify the mode of delivery. Preferably use “web-based” and/or “mobile” and/or “electronic game” in the title. Avoid ambiguous terms like “online”, “virtual”, “interactive”. Use “Internet-based” only if Intervention includes non-web-based Internet components (e.g. email), use “computer-based” or “electronic” only if offline products are used. Use “virtual” only in the context of “virtual reality” (3-D worlds). Use “online” only in the context of “online support groups”. Complement or substitute product names with broader terms for the class of products (such as “mobile” or “smart phone” instead of “iphone”), especially if the application runs on different platforms.

subitem not at all important

1 ( )

2 ( )

3 ( )

4 ( )

5 ( )

essential

Does your paper address subitem 1a-i? *

Copy and paste relevant sections from manuscript title (include quotes in quotation marks "like this" to indicate direct quotes from your manuscript), or elaborate on this item by providing additional information not in the ms, or briefly explain why the item is not applicable/relevant for your study

WeChat-Delivered Mobile Medical Nutrition Therapy Intervention in Gestational Diabetes Mellitus: A Randomized Controlled Trial

1a-ii) Non-web-based components or important co-interventions in title

Mention non-web-based components or important co-interventions in title, if any (e.g., “with telephone support”).

subitem not at all important

1 ○

2 ○

3 ○

4 ○

5 ○

essential

Does your paper address subitem 1a-ii?

Copy and paste relevant sections from manuscript title (include quotes in quotation marks "like this" to indicate direct quotes from your manuscript), or elaborate on this item by providing additional information not in the ms, or briefly explain why the item is not applicable/relevant for your study

您的回答

1a-iii) Primary condition or target group in the title

Mention primary condition or target group in the title, if any (e.g., “for children with Type I Diabetes”) Example: A Web-based and Mobile Intervention with Telephone Support for Children with Type I Diabetes: Randomized Controlled Trial

subitem not at all important

1 ○

2 ○

3 ○

4 ○

5 ○

essential

Does your paper address subitem 1a-iii? *

Copy and paste relevant sections from manuscript title (include quotes in quotation marks "like this" to indicate direct quotes from your manuscript), or elaborate on this item by providing additional information not in the ms, or briefly explain why the item is not applicable/relevant for your study

WeChat-Delivered Mobile Medical Nutrition Therapy Intervention in Gestational Diabetes Mellitus: A Randomized Controlled Trial

1b) ABSTRACT: Structured summary of trial design, methods, results, and conclusions

NPT extension: Description of experimental treatment, comparator, care providers, centers, and blinding status.

1b-i) Key features/functionalities/components of the intervention and comparator in the METHODS section of the ABSTRACT

Mention key features/functionalities/components of the intervention and comparator in the abstract. If possible, also mention theories and principles used for designing the site. Keep in mind the needs of systematic reviewers and indexers by including important synonyms. (Note: Only report in the abstract what the main paper is reporting. If this information is missing from the main body of text, consider adding it)

subitem not at all important

1 ○

2 ○

3 ○

4 ○

5 ○

essential

Does your paper address subitem 1b-i? *

Copy and paste relevant sections from the manuscript abstract (include quotes in quotation marks "like this" to indicate direct quotes from your manuscript), or elaborate on this item by providing additional information not in the ms, or briefly explain why the item is not applicable/relevant for your study

Methods: A parallel-group, single-blind randomized controlled trial (with outcome assessor blinding) was implemented at a university hospital's obstetric clinic. Eligible participants were those with a 24–28 week singleton pregnancy, a GDM diagnosis confirmed by the 75-g oral glucose tolerance test, no requirement for insulin therapy, and the ability to use a smartphone and WeChat, as well as communicate in Chinese. .. .. Intention-to-treat analysis using adjusted generalized linear mixed models was used to evaluate the effects of the intervention.
Results: From March 2023 to October 2023, a total of 94 participants were enrolled in this study. ...Compared with the control group, the intervention group showed a significantly greater decrease in GWG over time (group×time interaction: β = -1.96, 95% CI: -3.58 to -0.34; P = 0.02), with no significant effects on FPG. Conversely, significant decreases in 2hPG (β = -0.12, 95% CI: -0.19 to -0.04; P < 0.001) and HbA1c (β = -0.49, 95% CI: -0.74 to -0.23; P < 0.001) were noted in the intervention group.
Conclusions: As a WeChat-delivered, BCW-informed intervention, WeMNT successfully reduced 2hPG, HbA1c, and GWG in GDM management. Its integrated self-management model yields robust evidence, validating this patient-centered tool as a viable option for clinical adoption.

1b-ii) Level of human involvement in the METHODS section of the ABSTRACT

Clarify the level of human involvement in the abstract, e.g., use phrases like "fully automated" vs. "therapist/nurse/care provider/physician-assisted" (mention number and expertise of providers involved, if any). (Note: Only report in the abstract what the main paper is reporting. If this information is missing from the main body of text, consider adding it)

subitem not at all important

1 ○

2 ○

3 ○

4 ○

5 ○

Does your paper address subitem 1b-ii?

Copy and paste relevant sections from the manuscript abstract (include quotes in quotation marks "like this" to indicate direct quotes from your manuscript), or elaborate on this item by providing additional information not in the ms, or briefly explain why the item is not applicable/relevant for your study

您的回答

1b-iii) Open vs. closed, web-based (self-assessment) vs. face-to-face assessments in the METHODS section of the ABSTRACT

Mention how participants were recruited (online vs. offline), e.g., from an open access website or from a clinic or a closed online user group (closed usergroup trial), and clarify if this was a purely web-based trial, or there were face-to-face components (as part of the intervention or for assessment). Clearly say if outcomes were self-assessed through questionnaires (as common in web-based trials). Note: In traditional offline trials, an open trial (open-label trial) is a type of clinical trial in which both the researchers and participants know which treatment is being administered. To avoid confusion, use “blinded” or “unblinded” to indicated the level of blinding instead of “open”, as “open” in web-based trials usually refers to “open access” (i.e. participants can self-enrol). (Note: Only report in the abstract what the main paper is reporting. If this information is missing from the main body of text, consider adding it)

subitem not at all important

1 ○

2 ○

3 ○

4 ○

5 ○

essential

Does your paper address subitem 1b-iii?

Copy and paste relevant sections from the manuscript abstract (include quotes in quotation marks "like this" to indicate direct quotes from your manuscript), or elaborate on this item by providing additional information not in the ms, or briefly explain why the item is not applicable/relevant for your study

您的回答

1b-iv) RESULTS section in abstract must contain use data

Report number of participants enrolled/assessed in each group, the use/uptake of the intervention (e.g., attrition/adherence metrics, use over time, number of logins etc.), in addition to primary/secondary outcomes. (Note: Only report in the abstract what the main paper is reporting. If this information is missing from the main body of text, consider adding it)

subitem not at all important

1 ○

2 ○

3 ○

4 ○

5 ○

essential

Does your paper address subitem 1b-iv?

Copy and paste relevant sections from the manuscript abstract (include quotes in quotation marks "like this" to indicate direct quotes from your manuscript), or elaborate on this item by providing additional information not in the ms, or briefly explain why the item is not applicable/relevant for your study

您的回答

1b-v) CONCLUSIONS/DISCUSSION in abstract for negative trials

Conclusions/Discussions in abstract for negative trials: Discuss the primary outcome - if the trial is negative (primary outcome not changed), and the intervention was not used, discuss whether negative results are attributable to lack of uptake and discuss reasons. (Note: Only report in the abstract what the main paper is reporting. If this information is missing from the main body of text, consider adding it)

subitem not at all important

1 ○

2 ○

3 ○

4 ○

5 ○

essential

Does your paper address subitem 1b-v?

Copy and paste relevant sections from the manuscript abstract (include quotes in quotation marks "like this" to indicate direct quotes from your manuscript), or elaborate on this item by providing additional information not in the ms, or briefly explain why the item is not applicable/relevant for your study

您的回答

INTRODUCTION

2a) In INTRODUCTION: Scientific background and explanation of rationale

2a-i) Problem and the type of system/solution

Describe the problem and the type of system/solution that is object of the study: intended as stand-alone intervention vs. incorporated in broader health care program? Intended for a particular patient population? Goals of the intervention, e.g., being more cost-effective to other interventions, replace or complement other solutions? (Note: Details about the intervention are provided in “Methods” under 5)

subitem not at all important

1 ◯

2 ◯

3 ◯

4 ◯

5 ◯

essential

Does your paper address subitem 2a-i? *

Copy and paste relevant sections from the manuscript (include quotes in quotation marks "like this" to indicate direct quotes from your manuscript), or elaborate on this item by providing additional information not in the ms, or briefly explain why the item is not applicable/relevant for your study

A dietary plan is key to managing GDM, yet women often struggle to adopt and sustain new dietary habits and self-management behaviors [12]. Long-term adherence to complex dietary plans is challenging without continuous support [13]. Providing adequate self-management support empowers patients to control their diet and blood glucose levels. As they gain self-management skills, positive behavioral changes become evident [13]. ....Currently, various mHealth tools often do not fully address the needs of women with GDM [18]. Both patients and health care professionals have stressed the importance of improving usability, personalization, information adequacy, and communication functionalities [18]. These findings highlight the ongoing need to develop and optimize mHealth solutions to better support self-management among women with GDM....we developed WeMNT, a WeChat-delivered MNT program tailored for women with GDM. Based on clinical expertise and patient feedback, we hypothesize that WeMNT will improve the effectiveness of GDM management.

2a-ii) Scientific background, rationale: What is known about the (type of) system

Scientific background, rationale: What is known about the (type of) system that is the object of the study (be sure to discuss the use of similar systems for other conditions/diagnoses, if appropiate), motivation for the study, i.e. what are the reasons for and what is the context for this specific study, from which stakeholder viewpoint is the study performed, potential impact of findings [2]. Briefly justify the choice of the comparator.

subitem not at all important

1 ○

2 ○

3 ○

4 ○

5 ○

essential

Does your paper address subitem 2a-ii? *

Copy and paste relevant sections from the manuscript (include quotes in quotation marks "like this" to indicate direct quotes from your manuscript), or elaborate on this item by providing additional information not in the ms, or briefly explain why the item is not applicable/relevant for your study

Mobile health (mHealth) and digital interventions have become essential complementary tools for supporting women with GDM and health care professionals. Recent studies report an increasing reliance on digital platforms because of their flexibility and convenience, which help overcome limited clinical contact time and provide continuous, personalized support for self-management [16, 17]. Both women and health care professionals expressed openness to using technology as an adjunct to standard care but emphasized the need for improvements in usability, such as enhanced content layout, user interface design, and data visualization [17]. Participants also highlighted the importance of features supporting diverse data recording, personalization, comprehensive information delivery, and effective communication with health care providers and peer networks [17]. Currently, various mHealth tools often do not fully address the needs of women with GDM [18]. Both patients and health care professionals have stressed the importance of improving usability, personalization, information adequacy, and communication functionalities [18]. These findings highlight the ongoing need to develop and optimize mHealth solutions to better support self-management among women with GDM.

2b) In INTRODUCTION: Specific objectives or hypotheses

Does your paper address CONSORT subitem 2b? *

Copy and paste relevant sections from the manuscript (include quotes in quotation marks "like this" to indicate direct quotes from your manuscript), or elaborate on this item by providing additional information not in the ms, or briefly explain why the item is not applicable/relevant for your study

Building on the BCW framework, we developed WeMNT, a WeChat-delivered MNT program tailored for women with GDM. Based on clinical expertise and patient feedback, we hypothesize that WeMNT will improve the effectiveness of GDM management. This study aims to evaluate the benefits of WeMNT, a WeChat-delivered MNT intervention program developed from clinical knowledge and BCW, for women with GDM.

METHODS

3a) Description of trial design (such as parallel, factorial) including allocation ratio

Does your paper address CONSORT subitem 3a? *

Copy and paste relevant sections from the manuscript (include quotes in quotation marks "like this" to indicate direct quotes from your manuscript), or elaborate on this item by providing additional information not in the ms, or briefly explain why the item is not applicable/relevant for your study

This single-blind, parallel-group randomized controlled trial (RCT)—with blinding limited specifically to outcome assessors—was carried out at the obstetric clinic affiliated with a university hospital.

3b) Important changes to methods after trial commencement (such as eligibility criteria), with reasons

Does your paper address CONSORT subitem 3b? *

Copy and paste relevant sections from the manuscript (include quotes in quotation marks "like this" to indicate direct quotes from your manuscript), or elaborate on this item by providing additional information not in the ms, or briefly explain why the item is not applicable/relevant for your study

The inclusion criteria included singleton pregnancy at 24–28 weeks, GDM diagnosed by a 75 g oral glucose tolerance test (OGTT)[20], no insulin requirement, and the ability to use a smartphone with WeChat and communicate in Chinese.

3b-i) Bug fixes, Downtimes, Content Changes

Bug fixes, Downtimes, Content Changes: ehealth systems are often dynamic systems. A description of changes to methods therefore also includes important changes made on the intervention or comparator during the trial (e.g., major bug fixes or changes in the functionality or content) (5-iii) and other “unexpected events” that may have influenced study design such as staff changes, system failures/downtimes, etc. [2].

subitem not at all important

1 ○

2 ○

3 ○

4 ○

5 ○

essential

Does your paper address subitem 3b-i?

Copy and paste relevant sections from the manuscript (include quotes in quotation marks "like this" to indicate direct quotes from your manuscript), or elaborate on this item by providing additional information not in the ms, or briefly explain why the item is not applicable/relevant for your study

您的回答

4a) Eligibility criteria for participants

Does your paper address CONSORT subitem 4a? *

Copy and paste relevant sections from the manuscript (include quotes in quotation marks "like this" to indicate direct quotes from your manuscript), or elaborate on this item by providing additional information not in the ms, or briefly explain why the item is not applicable/relevant for your study

The inclusion criteria included singleton pregnancy at 24–28 weeks, GDM diagnosed by a 75 g oral glucose tolerance test (OGTT)[20], no insulin requirement, and the ability to use a smartphone with WeChat and communicate in Chinese.

4a-i) Computer / Internet literacy

Computer / Internet literacy is often an implicit “de facto” eligibility criterion - this should be explicitly clarified.

subitem not at all important

1 ○

2 ○

3 ○

4 ○

5 ○

essential

Does your paper address subitem 4a-i?

Copy and paste relevant sections from the manuscript (include quotes in quotation marks "like this" to indicate direct quotes from your manuscript), or elaborate on this item by providing additional information not in the ms, or briefly explain why the item is not applicable/relevant for your study

您的回答

4a-ii) Open vs. closed, web-based vs. face-to-face assessments:

Open vs. closed, web-based vs. face-to-face assessments: Mention how participants were recruited (online vs. offline), e.g., from an open access website or from a clinic, and clarify if this was a purely web-based trial, or there were face-to-face components (as part of the intervention or for assessment), i.e., to what degree got the study team to know the participant. In online-only trials, clarify if participants were quasi-anonymous and whether having multiple identities was possible or whether technical or logistical measures (e.g., cookies, email confirmation, phone calls) were used to detect/prevent these.

subitem not at all important

1

2

3

4

5

essential

Does your paper address subitem 4a-ii? *

Copy and paste relevant sections from the manuscript (include quotes in quotation marks "like this" to indicate direct quotes from your manuscript), or elaborate on this item by providing additional information not in the ms, or briefly explain why the item is not applicable/relevant for your study

Recruitment and Randomization
Participants were enrolled during their standard antenatal care appointments. Clinic physicians and nursing staff assisted in recruitment by identifying potential participants who met the eligibility criteria and had recently completed the OGTT. These health care providers also disseminated study information to these individuals.
Interested candidates underwent an initial eligibility assessment by the research team. A follow-up appointment was then scheduled at the clinic, typically within one week of the OGTT, to discuss the study in detail, obtain written informed consent, and initiate enrollment.
Following the provision of informed consent, eligible participants were randomly assigned to either the intervention group (n = 47) or the control group (n = 47) in a 1:1 ratio, with randomization sequences generated by a computer. To ensure allocation concealment, sequentially numbered, sealed, opaque envelopes were used. This process preserved the integrity of the randomization and minimized selection bias. The flow of participants throughout the study is detailed in Figure 1.

4a-iii) Information giving during recruitment

Information given during recruitment. Specify how participants were briefed for recruitment and in the informed consent procedures (e.g., publish the informed consent documentation as appendix, see also item X26), as this information may have an effect on user self-selection, user expectation and may also bias results.

subitem not at all important

1 ○

2 ○

3 ○

4 ○

5 ○

essential

Does your paper address subitem 4a-iii?

Copy and paste relevant sections from the manuscript (include quotes in quotation marks "like this" to indicate direct quotes from your manuscript), or elaborate on this item by providing additional information not in the ms, or briefly explain why the item is not applicable/relevant for your study

Recruitment and Randomization
Participants were enrolled during their standard antenatal care appointments. Clinic physicians and nursing staff assisted in recruitment by identifying potential participants who met the eligibility criteria and had recently completed the OGTT. These health care providers also disseminated study information to these individuals.
Interested candidates underwent an initial eligibility assessment by the research team. A follow-up appointment was then scheduled at the clinic, typically within one week of the OGTT, to discuss the study in detail, obtain written informed consent, and initiate enrollment.
Following the provision of informed consent, eligible participants were randomly assigned to either the intervention group (n = 47) or the control group (n = 47) in a 1:1 ratio, with randomization sequences generated by a computer. To ensure allocation concealment, sequentially numbered, sealed, opaque envelopes were used. This process preserved the integrity of the randomization and minimized selection bias. The flow of participants throughout the study is detailed in Figure 1.

4b) Settings and locations where the data were collected

Does your paper address CONSORT subitem 4b? *

Copy and paste relevant sections from the manuscript (include quotes in quotation marks "like this" to indicate direct quotes from your manuscript), or elaborate on this item by providing additional information not in the ms, or briefly explain why the item is not applicable/relevant for your study

Research nurses contacted participants by phone or WeChat to schedule data collection sessions, and all assessments were completed by participants in the obstetric clinic, with onsite researcher assistance available if needed. HbA1c was measured twice, specifically at baseline and at the endpoint. To prevent contamination between groups, data collection procedures for the intervention and control groups were scheduled on separate dates at the study site.

4b-i) Report if outcomes were (self-)assessed through online questionnaires

Clearly report if outcomes were (self-)assessed through online questionnaires (as common in web-based trials) or otherwise.

subitem not at all important

1 ○

2 ○

3 ○

4 ○

5 ○

essential

Does your paper address subitem 4b-i? *

Copy and paste relevant sections from the manuscript (include quotes in quotation marks "like this" to indicate direct quotes from your manuscript), or elaborate on this item by providing additional information not in the ms, or briefly explain why the item is not applicable/relevant for your study

outcomes were not (self-)assessed through online questionnaires

4b-ii) Report how institutional affiliations are displayed

Report how institutional affiliations are displayed to potential participants [on ehealth media], as affiliations with prestigious hospitals or universities may affect volunteer rates, use, and reactions with regards to an intervention.(Not a required item – describe only if this may bias results)

subitem not at all important

1 ○

2 ○

3 ○

4 ○

5 ○

essential

Does your paper address subitem 4b-ii?

Copy and paste relevant sections from the manuscript (include quotes in quotation marks "like this" to indicate direct quotes from your manuscript), or elaborate on this item by providing additional information not in the ms, or briefly explain why the item is not applicable/relevant for your study

您的回答

5) The interventions for each group with sufficient details to allow replication, including how and when they were actually administered

5-i) Mention names, credential, affiliations of the developers, sponsors, and owners

Mention names, credential, affiliations of the developers, sponsors, and owners [6] (if authors/evaluators are owners or developer of the software, this needs to be declared in a “Conflict of interest” section or mentioned elsewhere in the manuscript).

subitem not at all important

1 ○

2 ○

3 ○

4 ○

5 ○

essential

Does your paper address subitem 5-i?

Copy and paste relevant sections from the manuscript (include quotes in quotation marks "like this" to indicate direct quotes from your manuscript), or elaborate on this item by providing additional information not in the ms, or briefly explain why the item is not applicable/relevant for your study

In this mHealth study, we use well-established nonprofit public WeChat mini-programs as the intervention platform. First, the “Diet Diary” mini-program was developed by a certified dietitian, Zhongyi Gu, as a nonprofit public health tool aligned with the American Diabetes Association guidelines for GDM [5]

5-ii) Describe the history/development process

Describe the history/development process of the application and previous formative evaluations (e.g., focus groups, usability testing), as these will have an impact on adoption/use rates and help with interpreting results.

subitem not at all important

1 ○

2 ○

3 ○

4 ○

5 ○

essential

Does your paper address subitem 5-ii?

Copy and paste relevant sections from the manuscript (include quotes in quotation marks "like this" to indicate direct quotes from your manuscript), or elaborate on this item by providing additional information not in the ms, or briefly explain why the item is not applicable/relevant for your study

您的回答

5-iii) Revisions and updating

Revisions and updating. Clearly mention the date and/or version number of the application/intervention (and comparator, if applicable) evaluated, or describe whether the intervention underwent major changes during the evaluation process, or whether the development and/or content was “frozen” during the trial. Describe dynamic components such as news feeds or changing content which may have an impact on the replicability of the intervention (for unexpected events see item 3b).

subitem not at all important

1 ○

2 ○

3 ○

4 ○

5 ○

essential

Does your paper address subitem 5-iii?

Copy and paste relevant sections from the manuscript (include quotes in quotation marks "like this" to indicate direct quotes from your manuscript), or elaborate on this item by providing additional information not in the ms, or briefly explain why the item is not applicable/relevant for your study

您的回答

5-iv) Quality assurance methods

Provide information on quality assurance methods to ensure accuracy and quality of information provided [1], if applicable.

subitem not at all important

1

2

3

4

5

essential

Does your paper address subitem 5-iv?

Copy and paste relevant sections from the manuscript (include quotes in quotation marks "like this" to indicate direct quotes from your manuscript), or elaborate on this item by providing additional information not in the ms, or briefly explain why the item is not applicable/relevant for your study

您的回答

5-v) Ensure replicability by publishing the source code, and/or providing screenshots/screen-capture video, and/or providing flowcharts of the algorithms used

Ensure replicability by publishing the source code, and/or providing screenshots/screen-capture video, and/or providing flowcharts of the algorithms used. Replicability (i.e., other researchers should in principle be able to replicate the study) is a hallmark of scientific reporting.

subitem not at all important

1 ○

2 ○

3 ○

4 ○

5 ○

essential

Does your paper address subitem 5-v?

Copy and paste relevant sections from the manuscript (include quotes in quotation marks "like this" to indicate direct quotes from your manuscript), or elaborate on this item by providing additional information not in the ms, or briefly explain why the item is not applicable/relevant for your study

您的回答

5-vi) Digital preservation

Digital preservation: Provide the URL of the application, but as the intervention is likely to change or disappear over the course of the years; also make sure the intervention is archived (Internet Archive, webcitation.org, and/or publishing the source code or screenshots/videos alongside the article). As pages behind login screens cannot be archived, consider creating demo pages which are accessible without login.

subitem not at all important

1 ○

2 ○

3 ○

4 ○

5 ○

essential

Does your paper address subitem 5-vi?

Copy and paste relevant sections from the manuscript (include quotes in quotation marks "like this" to indicate direct quotes from your manuscript), or elaborate on this item by providing additional information not in the ms, or briefly explain why the item is not applicable/relevant for your study

您的回答

5-vii) Access

Access: Describe how participants accessed the application, in what setting/context, if they had to pay (or were paid) or not, whether they had to be a member of specific group. If known, describe how participants obtained “access to the platform and Internet” [1]. To ensure access for editors/reviewers/readers, consider to provide a “backdoor” login account or demo mode for reviewers/readers to explore the application (also important for archiving purposes, see vi).

subitem not at all important

1 ○

2 ○

3 ○

4 ○

5 ○

essential

Does your paper address subitem 5-vii? *

Copy and paste relevant sections from the manuscript (include quotes in quotation marks "like this" to indicate direct quotes from your manuscript), or elaborate on this item by providing additional information not in the ms, or briefly explain why the item is not applicable/relevant for your study

In this mHealth study, we use well-established nonprofit public WeChat mini-programs as the intervention platform.

5-viii) Mode of delivery, features/functionalities/components of the intervention and comparator, and the theoretical framework

Describe mode of delivery, features/functionalities/components of the intervention and comparator, and the theoretical framework [6] used to design them (instructional strategy [1], behaviour change techniques, persuasive features, etc., see e.g., [7, 8] for terminology). This includes an in-depth description of the content (including where it is coming from and who developed it) [1],” whether [and how] it is tailored to individual circumstances and allows users to track their progress and receive feedback” [6]. This also includes a description of communication delivery channels and – if computer-mediated communication is a component – whether communication was synchronous or asynchronous [6]. It also includes information on presentation strategies [1], including page design principles, average amount of text on pages, presence of hyperlinks to other resources, etc. [1].

subitem not at all important

1 ○

2 ○

3 ○

4 ○

5 ○

essential

Does your paper address subitem 5-viii? *

Copy and paste relevant sections from the manuscript (include quotes in quotation marks "like this" to indicate direct quotes from your manuscript), or elaborate on this item by providing additional information not in the ms, or briefly explain why the item is not applicable/relevant for your study

Intervention Features
In this mHealth study, we use well-established nonprofit public WeChat mini-programs as the intervention platform. First, the “Diet Diary” mini-program was developed by a certified dietitian, Zhongyi Gu, as a nonprofit public health tool aligned with the American Diabetes Association guidelines for GDM [5].
Intervention process
Upon enrollment, each participant received an individualized MNT prescription developed by a study dietitian using the “Diet Diary” mini-program, which incorporated personal parameters (age, gestational age, pBMI) and specified precise gram weights of food types for daily meals and snacks. Participants recorded all food and beverage intake (type and weight) via the same mini-program, with digital food scales provided to support accurate measurement, and used the "Food Charts" self-learning module to learn about food selection, GI values, and portion control techniques. Concurrently, women logged blood glucose and body weight data into the “Pregnancy Sugar Weight Record” mini-program for continuous health monitoring. Those unable to input data independently could use paper records, audio/video recordings, or photos, with nurses assisting with data entry.

5-ix) Describe use parameters

Describe use parameters (e.g., intended “doses” and optimal timing for use). Clarify what instructions or recommendations were given to the user, e.g., regarding timing, frequency, heaviness of use, if any, or was the intervention used ad libitum.

subitem not at all important

1 ○

2 ○

3 ○

4 ○

5 ○

essential

Does your paper address subitem 5-ix?

Copy and paste relevant sections from the manuscript (include quotes in quotation marks "like this" to indicate direct quotes from your manuscript), or elaborate on this item by providing additional information not in the ms, or briefly explain why the item is not applicable/relevant for your study

Concurrently, women logged blood glucose and body weight data weekly into the “Pregnancy Sugar Weight Record” mini-program for continuous health monitoring. Those unable to input data independently could use paper records, audio/video recordings, or photos, with nurses assisting with data entry.

5-x) Clarify the level of human involvement

Clarify the level of human involvement (care providers or health professionals, also technical assistance) in the e-intervention or as co-intervention (detail number and expertise of professionals involved, if any, as well as “type of assistance offered, the timing and frequency of the support, how it is initiated, and the medium by which the assistance is delivered”. It may be necessary to distinguish between the level of human involvement required for the trial, and the level of human involvement required for a routine application outside of a RCT setting (discuss under item 21 – generalizability).

subitem not at all important

1 ○

2 ○

3 ○

4 ○

5 ○

essential

Does your paper address subitem 5-x?

Copy and paste relevant sections from the manuscript (include quotes in quotation marks "like this" to indicate direct quotes from your manuscript), or elaborate on this item by providing additional information not in the ms, or briefly explain why the item is not applicable/relevant for your study

A multidisciplinary team (certified dietitians, nurses, and obstetricians) provided comprehensive support throughout the study. Diabetes nurses reviewed participants' dietary logs, blood glucose levels, and weight records weekly via the WeChat backend, delivering personalized feedback and evaluations through WeChat messages or voice calls—acknowledging adherence achievements, addressing inquiries, and offering constructive suggestions.

5-xi) Report any prompts/reminders used

Report any prompts/reminders used: Clarify if there were prompts (letters, emails, phone calls, SMS) to use the application, what triggered them, frequency etc. It may be necessary to distinguish between the level of prompts/reminders required for the trial, and the level of prompts/reminders for a routine application outside of a RCT setting (discuss under item 21 – generalizability).

subitem not at all important

1 ○

2 ○

3 ○

4 ○

5 ○

essential

Does your paper address subitem 5-xi? *

Copy and paste relevant sections from the manuscript (include quotes in quotation marks "like this" to indicate direct quotes from your manuscript), or elaborate on this item by providing additional information not in the ms, or briefly explain why the item is not applicable/relevant for your study

For participants with persistently unmet glycemic targets, nurses collaborated with dietitians to analyze issues and adjust meal plans (e.g., modifying carbohydrate distribution or introducing alternative foods). A real-time consultation channel ensured that dietary inquiries received responses within 12 hours, and the team routinely audited 20% of the records to maintain intervention adherence standards. A nurse-modified WeChat peer support group facilitated weekly discussions in which women exchanged experiences, practical strategies, and insights into glycemic management and MNT implementation, fostering mutual encouragement. For participants facing persistent glucose management challenges for more than two weeks, the care team arranged 15–30 minutes of individual video consultations to provide focused support; these sessions could also lead to referrals for further multidisciplinary evaluation if needed. Individualized WeChat communications (messages, voice recordings, and video calls) were additionally provided for participants with specific needs, offering one-on-one dietary consultation and guidance. Throughout the intervention, all communications maintained an empathetic, nonjudgmental tone, and participants retained the flexibility to adjust their involvement or pause monitoring as needed. See Figure 2 for the implementation process of the study.

5-xii) Describe any co-interventions (incl. training/support)

Describe any co-interventions (incl. training/support): Clearly state any interventions that are provided in addition to the targeted eHealth intervention, as ehealth intervention may not be designed as stand-alone intervention. This includes training sessions and support [1]. It may be necessary to distinguish between the level of training required for the trial, and the level of training for a routine application outside of a RCT setting (discuss under item 21 – generalizability.

subitem not at all important

1 ○

2 ○

3 ○

4 ○

5 ○

essential

Does your paper address subitem 5-xii? *

Copy and paste relevant sections from the manuscript (include quotes in quotation marks "like this" to indicate direct quotes from your manuscript), or elaborate on this item by providing additional information not in the ms, or briefly explain why the item is not applicable/relevant for your study

For participants facing persistent glucose management challenges for more than two weeks, the care team arranged 15–30 minutes of individual video consultations to provide focused support; these sessions could also lead to referrals for further multidisciplinary evaluation if needed. Individualized WeChat communications (messages, voice recordings, and video calls) were additionally provided for participants with specific needs, offering one-on-one dietary consultation and guidance. Throughout the intervention, all communications maintained an empathetic, nonjudgmental tone, and participants retained the flexibility to adjust their involvement or pause monitoring as needed. See Figure 2 for the implementation process of the study.

6a) Completely defined pre-specified primary and secondary outcome measures, including how and when they were assessed

Does your paper address CONSORT subitem 6a? *

Copy and paste relevant sections from the manuscript (include quotes in quotation marks "like this" to indicate direct quotes from your manuscript), or elaborate on this item by providing additional information not in the ms, or briefly explain why the item is not applicable/relevant for your study

Outcome Measures
The primary outcome measures of this study included glycemic control status and weight changes during pregnancy. Glycemic control is assessed using FPG and 2hPG levels, both of which are measured on a Hitachi 7600 analyzer using venous plasma samples. Additionally, HbA1c is determined through high-performance liquid chromatography. Gestational weight gain (GWG) was defined as the weight accrued during pregnancy and was computed as the numerical difference between the measured weight values at each time point (baseline and subsequent follow-up) and the self-reported prepregnancy weight. Secondary outcome measures include obstetric complications and neonatal outcomes, such as gestational hypertension, preterm birth (< 37 weeks) and neonatal birth weight. The amniotic fluid index (AFI) was included as a secondary outcome because of its reported association with GDM-related fetal oligohydramnios and polyhydramnios, which may increase the risks of preterm labor and cesarean delivery. AFI was measured by ultrasound using the four-quadrant technique after the 36th week and prior to delivery, with oligohydramnios defined as an AFI < 5 cm and polyhydramnios as an AFI > 25 cm, respectively. All measurements are conducted in strict adherence to standardized clinical practices.

6a-i) Online questionnaires: describe if they were validated for online use and apply CHERRIES items to describe how the questionnaires were designed/deployed

If outcomes were obtained through online questionnaires, describe if they were validated for online use and apply CHERRIES items to describe how the questionnaires were designed/deployed [9].

subitem not at all important

1 ◯

2 ◯

3 ◯

4 ◯

5 ◯

essential

Does your paper address subitem 6a-i?

Copy and paste relevant sections from manuscript text

您的回答

6a-ii) Describe whether and how “use” (including intensity of use/dosage) was defined/measured/monitored

Describe whether and how “use” (including intensity of use/dosage) was defined/measured/monitored (logins, logfile analysis, etc.). Use/adoption metrics are important process outcomes that should be reported in any ehealth trial.

subitem not at all important

1 ○

2 ○

3 ○

4 ○

5 ○

essential

Does your paper address subitem 6a-ii?

Copy and paste relevant sections from manuscript text

Outcome Measures
The primary outcome measures of this study included glycemic control status and weight changes during pregnancy. Glycemic control is assessed using FPG and 2hPG levels, both of which are measured on a Hitachi 7600 analyzer using venous plasma samples. Additionally, HbA1c is determined through high-performance liquid chromatography. Gestational weight gain (GWG) was defined as the weight accrued during pregnancy and was computed as the numerical difference between the measured weight values at each time point (baseline and subsequent follow-up) and the self-reported prepregnancy weight. Secondary outcome measures include obstetric complications and neonatal outcomes, such as gestational hypertension, preterm birth (< 37 weeks) and neonatal birth weight. The amniotic fluid index (AFI) was included as a secondary outcome because of its reported association with GDM-related fetal oligohydramnios and polyhydramnios, which may increase the risks of preterm labor and cesarean delivery. AFI was measured by ultrasound using the four-quadrant technique after the 36th week and prior to delivery, with oligohydramnios defined as an AFI < 5 cm and polyhydramnios as an AFI > 25 cm, respectively. All measurements are conducted in strict adherence to standardized clinical practices.

6a-iii) Describe whether, how, and when qualitative feedback from participants was obtained

Describe whether, how, and when qualitative feedback from participants was obtained (e.g., through emails, feedback forms, interviews, focus groups).

subitem not at all important

1 ○

2 ○

3 ○

4 ○

5 ○

essential

Does your paper address subitem 6a-iii?

Copy and paste relevant sections from manuscript text

您的回答

6b) Any changes to trial outcomes after the trial commenced, with reasons

Does your paper address CONSORT subitem 6b? *

Copy and paste relevant sections from the manuscript (include quotes in quotation marks "like this" to indicate direct quotes from your manuscript), or elaborate on this item by providing additional information not in the ms, or briefly explain why the item is not applicable/relevant for your study

no changes to trial outcomes after the trial commenced

7a) How sample size was determined

NPT: When applicable, details of whether and how the clustering by care provides or centers was addressed

7a-i) Describe whether and how expected attrition was taken into account when calculating the sample size

Describe whether and how expected attrition was taken into account when calculating the sample size.

subitem not at all important

1 ○

2 ○

3 ○

4 ○

5 ○

essential

Does your paper address subitem 7a-i?

Copy and paste relevant sections from manuscript title (include quotes in quotation marks "like this" to indicate direct quotes from your manuscript), or elaborate on this item by providing additional information not in the ms, or briefly explain why the item is not applicable/relevant for your study

Sample Size Calculation
The study was powered on the primary outcome of HbA1c. To determine the appropriate sample size, we first identified the effect size based on the relevant literature. A meta-analysis revealed that telemedicine interventions for women with GDM yielded an effect size of .63 (Cohen's *d*, i.e., a standardized mean difference between groups) for HbA1c reduction; specifically, studies utilizing WeChat as the intervention tool (6 RCTs in total) demonstrated a greater effect size of .84 for HbA1c improvement [21]. Considering both clinical relevance and methodological conservatism, we determined a minimum effect size (Cohen's d) of .65 for the sample size calculation.
GPower 3.1 software was employed to perform the a priori sample size calculation using the following key parameters: effect size (d) = .65, significance level (α) = .05 (two-tailed), and statistical power (1-β) = .80. The calculation results indicated a basic sample size of 78 participants. To address a potential 15% attrition rate, a common consideration in longitudinal clinical studies, the final sample size was adjusted to 94 participants, which ensures adequate statistical power for detecting the prespecified effect on the primary outcome (HbA1c).

7b) When applicable, explanation of any interim analyses and stopping guidelines

Does your paper address CONSORT subitem 7b? *

Copy and paste relevant sections from the manuscript (include quotes in quotation marks "like this" to indicate direct quotes from your manuscript), or elaborate on this item by providing additional information not in the ms, or briefly explain why the item is not applicable/relevant for your study

The study endpoint was defined as delivery, including preterm delivery (consistent with clinical criteria, i.e., delivery between the 28th and 37th gestational weeks) and term delivery (delivery at or after the 38th gestational week). Participants with preterm delivery may miss the follow-up assessments scheduled thereafter and reach the study endpoint ahead of schedule (early closure) compared to term delivery (routine closure).

8a) Method used to generate the random allocation sequence

NPT: When applicable, how care providers were allocated to each trial group

Does your paper address CONSORT subitem 8a? *

Copy and paste relevant sections from the manuscript (include quotes in quotation marks "like this" to indicate direct quotes from your manuscript), or elaborate on this item by providing additional information not in the ms, or briefly explain why the item is not applicable/relevant for your study

Following the provision of informed consent, eligible participants were randomly assigned to either the intervention group (n = 47) or the control group (n = 47) in a 1:1 ratio, with randomization sequences generated by a computer. To ensure allocation concealment, sequentially numbered, sealed, opaque envelopes were used. This process preserved the integrity of the randomization and minimized selection bias.

8b) Type of randomisation; details of any restriction (such as blocking and block size)

Does your paper address CONSORT subitem 8b? *

Copy and paste relevant sections from the manuscript (include quotes in quotation marks "like this" to indicate direct quotes from your manuscript), or elaborate on this item by providing additional information not in the ms, or briefly explain why the item is not applicable/relevant for your study

Following the provision of informed consent, eligible participants were randomly assigned to either the intervention group (n = 47) or the control group (n = 47) in a 1:1 ratio, with randomization sequences generated by a computer. To ensure allocation concealment, sequentially numbered, sealed, opaque envelopes were used. This process preserved the integrity of the randomization and minimized selection bias.

9) Mechanism used to implement the random allocation sequence (such as sequentially numbered containers), describing any steps taken to conceal the sequence until interventions were assigned

Does your paper address CONSORT subitem 9? *

Copy and paste relevant sections from the manuscript (include quotes in quotation marks "like this" to indicate direct quotes from your manuscript), or elaborate on this item by providing additional information not in the ms, or briefly explain why the item is not applicable/relevant for your study

Following the provision of informed consent, eligible participants were randomly assigned to either the intervention group (n = 47) or the control group (n = 47) in a 1:1 ratio, with randomization sequences generated by a computer. To ensure allocation concealment, sequentially numbered, sealed, opaque envelopes were used. This process preserved the integrity of the randomization and minimized selection bias.

10) Who generated the random allocation sequence, who enrolled participants, and who assigned participants to interventions

Does your paper address CONSORT subitem 10? *

Copy and paste relevant sections from the manuscript (include quotes in quotation marks "like this" to indicate direct quotes from your manuscript), or elaborate on this item by providing additional information not in the ms, or briefly explain why the item is not applicable/relevant for your study

Following the provision of informed consent, eligible participants were randomly assigned to either the intervention group (n = 47) or the control group (n = 47) in a 1:1 ratio, with randomization sequences generated by a computer. To ensure allocation concealment, sequentially numbered, sealed, opaque envelopes were used. This process preserved the integrity of the randomization and minimized selection bias.

11a) If done, who was blinded after assignment to interventions (for example, participants, care providers, those assessing outcomes) and how

NPT: Whether or not administering co-interventions were blinded to group assignment

11a-i) Specify who was blinded, and who wasn’t

Specify who was blinded, and who wasn’t. Usually, in web-based trials it is not possible to blind the participants [1, 3] (this should be clearly acknowledged), but it may be possible to blind outcome assessors, those doing data analysis or those administering co-interventions (if any).

subitem not at all important

1 ○

2 ○

3 ○

4 ○

5 ○

essential

Does your paper address subitem 11a-i? *

Copy and paste relevant sections from the manuscript (include quotes in quotation marks "like this" to indicate direct quotes from your manuscript), or elaborate on this item by providing additional information not in the ms, or briefly explain why the item is not applicable/relevant for your study

Data collection was performed by research nurses who were blinded to group allocation

11a-ii) Discuss e.g., whether participants knew which intervention was the “intervention of interest” and which one was the “comparator”

Informed consent procedures (4a-ii) can create biases and certain expectations - discuss e.g., whether participants knew which intervention was the “intervention of interest” and which one was the “comparator”.

subitem not at all important

1 ○

2 ○

3 ○

4 ○

5 ○

essential

Does your paper address subitem 11a-ii?

Copy and paste relevant sections from the manuscript (include quotes in quotation marks "like this" to indicate direct quotes from your manuscript), or elaborate on this item by providing additional information not in the ms, or briefly explain why the item is not applicable/relevant for your study

您的回答

11b) If relevant, description of the similarity of interventions

(this item is usually not relevant for ehealth trials as it refers to similarity of a placebo or sham intervention to a active medication/intervention)

Does your paper address CONSORT subitem 11b? *

Copy and paste relevant sections from the manuscript (include quotes in quotation marks "like this" to indicate direct quotes from your manuscript), or elaborate on this item by providing additional information not in the ms, or briefly explain why the item is not applicable/relevant for your study

See Table 2 for a detailed comparison of management approaches across modules between the WeMNT intervention group and control group.

12a) Statistical methods used to compare groups for primary and secondary outcomes

NPT: When applicable, details of whether and how the clustering by care providers or centers was addressed

Does your paper address CONSORT subitem 12a? *

Copy and paste relevant sections from the manuscript (include quotes in quotation marks "like this" to indicate direct quotes from your manuscript), or elaborate on this item by providing additional information not in the ms, or briefly explain why the item is not applicable/relevant for your study

Generalized linear mixed models (GLMMs) were used to assess the intervention's effects on primary outcomes (FPG, 2hPG, and GWG) across different time points: 4 (time: baseline, 1 month, 2 months, and endpoint) × 2 (group: intervention vs. control). General sociodemographic characteristics and blood glucose management-related data were incorporated as covariates in this model to control for potential confounding factors.
Additionally, a distinct 2 (time: baseline and endpoint) × 2 (group: intervention vs. control) GLMM was established to examine longitudinal changes in HbA1c levels and to compare between-group differences in HbA1c.
Secondary outcomes (maternal and fetal clinical measures) were analyzed using per-protocol methods. For the assessment of between-group disparities, we utilized independent-samples t-tests to compare continuous variables (e.g., AFI) and chi-square (χ^2) tests to analyze categorical variables such as the incidence of preeclampsia.

12a-i) Imputation techniques to deal with attrition / missing values

Imputation techniques to deal with attrition / missing values: Not all participants will use the intervention/comparator as intended and attrition is typically high in ehealth trials. Specify how participants who did not use the application or dropped out from the trial were treated in the statistical analysis (a complete case analysis is strongly discouraged, and simple imputation techniques such as LOCF may also be problematic [4]).

subitem not at all important

1 ○

2 ○

3 ○

4 ○

5 ○

essential

Does your paper address subitem 12a-i? *

Copy and paste relevant sections from the manuscript (include quotes in quotation marks "like this" to indicate direct quotes from your manuscript), or elaborate on this item by providing additional information not in the ms, or briefly explain why the item is not applicable/relevant for your study

Little’s missing completely at random (MCAR) tests were performed on the post-intervention outcome variables. The result was not statistically significant ($p = 0.1723 > 0.05$), indicating that the missing data mechanism is completely at random....GLMM was established to examine longitudinal changes in HbA1c levels and to compare between-group differences in HbA1c. This analytical approach was selected because it can account for within-subject correlations and manage missing data under the missing-at-random assumption, enabling the use of all available data points without the need for multiple imputation.

12b) Methods for additional analyses, such as subgroup analyses and adjusted analyses

Does your paper address CONSORT subitem 12b? *

Copy and paste relevant sections from the manuscript (include quotes in quotation marks "like this" to indicate direct quotes from your manuscript), or elaborate on this item by providing additional information not in the ms, or briefly explain why the item is not applicable/relevant for your study

This study has no subgroup analyses and adjusted analyses.

X26) REB/IRB Approval and Ethical Considerations [recommended as subheading under "Methods"] (not a CONSORT item)

X26-i) Comment on ethics committee approval

subitem not at all important

1 ○

2 ○

3 ○

4 ○

5 ○

essential

Does your paper address subitem X26-i?

Copy and paste relevant sections from the manuscript (include quotes in quotation marks "like this" to indicate direct quotes from your manuscript), or elaborate on this item by providing additional information not in the ms, or briefly explain why the item is not applicable/relevant for your study

Ethical Considerations
This study was approved by the hospital institutional ethics committee (Approval No. 2023-109-02) and abided by the principles of Declaration of Helsinki. All patients who participated in the study participated voluntarily and obtained the informed consent of each patient in writing. Patients have the right to withdraw from the study at any time. All exported data must undergo anonymization by the data manager before statistical analysis can be conducted. Individuals cannot be identified in any results presentation. No financial compensation was provided to avoid coercion.

x26-ii) Outline informed consent procedures

Outline informed consent procedures e.g., if consent was obtained offline or online (how? Checkbox, etc.?), and what information was provided (see 4a-ii). See [6] for some items to be included in informed consent documents.

subitem not at all important

1 ○

2 ○

3 ○

4 ○

5 ○

essential

Does your paper address subitem X26-ii?

Copy and paste relevant sections from the manuscript (include quotes in quotation marks "like this" to indicate direct quotes from your manuscript), or elaborate on this item by providing additional information not in the ms, or briefly explain why the item is not applicable/relevant for your study

Interested candidates underwent an initial eligibility assessment by the research team. A follow-up appointment was then scheduled at the clinic, typically within one week of the OGTT, to discuss the study in detail, obtain written informed consent, and initiate enrollment.

X26-iii) Safety and security procedures

Safety and security procedures, incl. privacy considerations, and any steps taken to reduce the likelihood or detection of harm (e.g., education and training, availability of a hotline)

subitem not at all important

1 ○

2 ○

3 ○

4 ○

5 ○

essential

Does your paper address subitem X26-iii?

Copy and paste relevant sections from the manuscript (include quotes in quotation marks "like this" to indicate direct quotes from your manuscript), or elaborate on this item by providing additional information not in the ms, or briefly explain why the item is not applicable/relevant for your study

您的回答

RESULTS

13a) For each group, the numbers of participants who were randomly assigned, received intended treatment, and were analysed for the primary outcome
NPT: The number of care providers or centers performing the intervention in each group and the number of patients treated by each care provider in each center

Does your paper address CONSORT subitem 13a? *

Copy and paste relevant sections from the manuscript (include quotes in quotation marks "like this" to indicate direct quotes from your manuscript), or elaborate on this item by providing additional information not in the ms, or briefly explain why the item is not applicable/relevant for your study

Results
Between March 2023 and October 2023, a total of 94 participants were recruited for this study. Among them, 88 participants (mean age: 32.18 years; SD: 5.04 years) successfully completed the study: 44 participants in the intervention group and 44 in the control group.

13b) For each group, losses and exclusions after randomisation, together with reasons

Does your paper address CONSORT subitem 13b? (NOTE: Preferably, this is shown in a CONSORT flow diagram) *

Copy and paste relevant sections from the manuscript (include quotes in quotation marks "like this" to indicate direct quotes from your manuscript), or elaborate on this item by providing additional information not in the ms, or briefly explain why the item is not applicable/relevant for your study

Results
Between March 2023 and October 2023, a total of 94 participants were recruited for this study. Among them, 88 participants (mean age: 32.18 years; SD: 5.04 years) successfully completed the study: 44 participants in the intervention group and 44 in the control group.
The participant flow and reasons for dropout are shown in Figure 1.

13b-i) Attrition diagram

Strongly recommended: An attrition diagram (e.g., proportion of participants still logging in or using the intervention/comparator in each group plotted over time, similar to a survival curve) or other figures or tables demonstrating usage/dose/engagement.

subitem not at all important

1 ○

2 ○

3 ○

4 ○

5 ○

essential

Does your paper address subitem 13b-i?

Copy and paste relevant sections from the manuscript or cite the figure number if applicable (include quotes in quotation marks "like this" to indicate direct quotes from your manuscript), or elaborate on this item by providing additional information not in the ms, or briefly explain why the item is not applicable/relevant for your study

The participant flow and reasons for dropout are shown in Figure 1.

14a) Dates defining the periods of recruitment and follow-up

Does your paper address CONSORT subitem 14a? *

Copy and paste relevant sections from the manuscript (include quotes in quotation marks "like this" to indicate direct quotes from your manuscript), or elaborate on this item by providing additional information not in the ms, or briefly explain why the item is not applicable/relevant for your study

Baseline data were collected during the OGTT, which was conducted between the 24th and 28th gestational weeks, and the intervention was initiated at the 28th gestational week. Two intermediate follow-up assessments were planned from intervention initiation to delivery, scheduled at the 32nd (1-month follow-up) and 36th weeks (2-month follow-up) , respectively. The study endpoint was defined as delivery, including preterm delivery (consistent with clinical criteria, i.e., delivery between the 28th and 37th gestational weeks) and term delivery (delivery at or after the 38th gestational week). Participants with preterm delivery may miss the follow-up assessments scheduled thereafter and reach the study endpoint ahead of schedule (early closure) compared to term delivery (routine closure).

14a-i) Indicate if critical “secular events” fell into the study period

Indicate if critical “secular events” fell into the study period, e.g., significant changes in Internet resources available or “changes in computer hardware or Internet delivery resources”

subitem not at all important

1 ○

2 ○

3 ○

4 ○

5 ○

essential

Does your paper address subitem 14a-i?

Copy and paste relevant sections from the manuscript (include quotes in quotation marks "like this" to indicate direct quotes from your manuscript), or elaborate on this item by providing additional information not in the ms, or briefly explain why the item is not applicable/relevant for your study

您的回答

14b) Why the trial ended or was stopped (early)

Does your paper address CONSORT subitem 14b? *

Copy and paste relevant sections from the manuscript (include quotes in quotation marks "like this" to indicate direct quotes from your manuscript), or elaborate on this item by providing additional information not in the ms, or briefly explain why the item is not applicable/relevant for your study

The study endpoint was defined as delivery, including preterm delivery (consistent with clinical criteria, i.e., delivery between the 28th and 37th gestational weeks) and term delivery (delivery at or after the 38th gestational week). Participants with preterm delivery may miss the follow-up assessments scheduled thereafter and reach the study endpoint ahead of schedule (early closure) compared to term delivery (routine closure).

15) A table showing baseline demographic and clinical characteristics for each group

NPT: When applicable, a description of care providers (case volume, qualification, expertise, etc.) and centers (volume) in each group

Does your paper address CONSORT subitem 15? *

Copy and paste relevant sections from the manuscript (include quotes in quotation marks "like this" to indicate direct quotes from your manuscript), or elaborate on this item by providing additional information not in the ms, or briefly explain why the item is not applicable/relevant for your study

Table 3. Baseline data across the intervention and control groups.

15-i) Report demographics associated with digital divide issues

In ehealth trials it is particularly important to report demographics associated with digital divide issues, such as age, education, gender, social-economic status, computer/Internet/ehealth literacy of the participants, if known.

subitem not at all important

1 ○

2 ○

3 ○

4 ○

5 ○

essential

Does your paper address subitem 15-i? *

Copy and paste relevant sections from the manuscript (include quotes in quotation marks "like this" to indicate direct quotes from your manuscript), or elaborate on this item by providing additional information not in the ms, or briefly explain why the item is not applicable/relevant for your study

see Table 3. Baseline data across the intervention and control groups.

16) For each group, number of participants (denominator) included in each analysis and whether the analysis was by original assigned groups

16-i) Report multiple “denominators” and provide definitions

Report multiple “denominators” and provide definitions: Report N’s (and effect sizes) “across a range of study participation [and use] thresholds” [1], e.g., N exposed, N consented, N used more than x times, N used more than y weeks, N participants “used” the intervention/comparator at specific pre-defined time points of interest (in absolute and relative numbers per group). Always clearly define “use” of the intervention.

subitem not at all important

1 ○

2 ○

3 ○

4 ○

5 ○

essential

Does your paper address subitem 16-i? *

Copy and paste relevant sections from the manuscript (include quotes in quotation marks "like this" to indicate direct quotes from your manuscript), or elaborate on this item by providing additional information not in the ms, or briefly explain why the item is not applicable/relevant for your study

The back-end records of the WeChat application show that in the intervention group, 87.2% (41 of 47) of the participants used the 'Diet Diary' mini-program for daily dietary logging, whereas 78.7% (37 of 47) employed the 'Pregnancy Sugar Weight Record' and 'Fetal Weight Assessor' for weight tracking. For the remaining participants who encountered difficulties inputting the data themselves, nurses assisted in entering the information into the mini-programs. All the participants explored the “food charts” module, learning the GI values of 476 foods, food selection and weighing. Initially, they engaged with these tools 2–3 times daily for 20–45 minutes, but usage decreased as they became more familiar with food attributes. They only revisited the information when needed. Among the 44 participants who completed the study, 54.5% (24 of 44) adhered to the diet plan ≥ 80% of the days, 13.6% (6 of 44) followed it for 60–79% of the days, 11.4% (5 of 44) for 40–59% of the days, another 11.4% (5 of 44) for 20–39% of the days, and 9.1% (4 of 44) for less than 20% of the days. In the control group, all 44 participants who completed the study reported independently studying the provided materials and following the dietary guidance for 60% - 80% of the days.

16-ii) Primary analysis should be intent-to-treat

Primary analysis should be intent-to-treat, secondary analyses could include comparing only “users”, with the appropriate caveats that this is no longer a randomized sample (see 18-i).

subitem not at all important

1 ○

2 ○

3 ○

4 ○

5 ○

essential

Does your paper address subitem 16-ii?

Copy and paste relevant sections from the manuscript (include quotes in quotation marks "like this" to indicate direct quotes from your manuscript), or elaborate on this item by providing additional information not in the ms, or briefly explain why the item is not applicable/relevant for your study

The primary efficacy analysis adhered to the intention-to-treat (ITT) principle, wherein all randomized participants (n = 94) were analyzed according to their original group assignments—this included individuals who were excluded during the study or lost to follow-up.

17a) For each primary and secondary outcome, results for each group, and the estimated effect size and its precision (such as 95% confidence interval)

Does your paper address CONSORT subitem 17a? *

Copy and paste relevant sections from the manuscript (include quotes in quotation marks "like this" to indicate direct quotes from your manuscript), or elaborate on this item by providing additional information not in the ms, or briefly explain why the item is not applicable/relevant for your study

Adjusted GLMMs with intention-to-treat (ITT) analysis revealed a significant group×time interaction for GWG (Table 5); notably, the intervention group achieved a significantly greater reduction in GWG over time compared with the control group, as indicated by the negative coefficient of the group×time interaction (β = -1.96, 95% CI: -3.58 to -0.34; P = .02). For FPG, no significant group×time interaction effect was observed. In contrast, both 2hPG and HbA1c showed significant group×time interaction effects. Compared with the control group, the intervention group demonstrated substantially greater decreases in 2hPG (β = -0.12, 95% CI: -0.19 to -0.04; P < .001) and HbA1c (β = -0.49, 95% CI: -0.74 to -0.23; P < .001) over time. The results of the per-protocol analyses (Supplementary Material 1) are consistent in terms of the direction and significance of the ITT analyses.

17a-i) Presentation of process outcomes such as metrics of use and intensity of use

In addition to primary/secondary (clinical) outcomes, the presentation of process outcomes such as metrics of use and intensity of use (dose, exposure) and their operational definitions is critical. This does not only refer to metrics of attrition (13-b) (often a binary variable), but also to more continuous exposure metrics such as “average session length”. These must be accompanied by a technical description how a metric like a “session” is defined (e.g., timeout after idle time) [1] (report under item 6a).

subitem not at all important

1 ○

2 ○

3 ○

4 ○

5 ○

essential

Does your paper address subitem 17a-i?

Copy and paste relevant sections from the manuscript (include quotes in quotation marks "like this" to indicate direct quotes from your manuscript), or elaborate on this item by providing additional information not in the ms, or briefly explain why the item is not applicable/relevant for your study

The back-end records of the WeChat application show that in the intervention group, 87.2% (41 of 47) of the participants used the 'Diet Diary' mini-program for daily dietary logging, whereas 78.7% (37 of 47) employed the 'Pregnancy Sugar Weight Record' and 'Fetal Weight Assessor' for weight tracking. For the remaining participants who encountered difficulties inputting the data themselves, nurses assisted in entering the information into the mini-programs. All the participants explored the “food charts” module, learning the GI values of 476 foods, food selection and weighing. Initially, they engaged with these tools 2–3 times daily for 20–45 minutes, but usage decreased as they became more familiar with food attributes. They only revisited the information when needed. Among the 44 participants who completed the study, 54.5% (24 of 44) adhered to the diet plan ≥ 80% of the days, 13.6% (6 of 44) followed it for 60–79% of the days, 11.4% (5 of 44) for 40–59% of the days, another 11.4% (5 of 44) for 20–39% of the days, and 9.1% (4 of 44) for less than 20% of the days. In the control group, all 44 participants who completed the study reported independently studying the provided materials and following the dietary guidance for 60% - 80% of the days.

17b) For binary outcomes, presentation of both absolute and relative effect sizes is recommended

Does your paper address CONSORT subitem 17b? *

Copy and paste relevant sections from the manuscript (include quotes in quotation marks "like this" to indicate direct quotes from your manuscript), or elaborate on this item by providing additional information not in the ms, or briefly explain why the item is not applicable/relevant for your study

There are no binary variables among the primary outcomes of the study.

18) Results of any other analyses performed, including subgroup analyses and adjusted analyses, distinguishing pre-specified from exploratory

Does your paper address CONSORT subitem 18? *

Copy and paste relevant sections from the manuscript (include quotes in quotation marks "like this" to indicate direct quotes from your manuscript), or elaborate on this item by providing additional information not in the ms, or briefly explain why the item is not applicable/relevant for your study

This study has no subgroup analyses and adjusted analyses, distinguishing pre-specified from exploratory

18-i) Subgroup analysis of comparing only users

A subgroup analysis of comparing only users is not uncommon in ehealth trials, but if done, it must be stressed that this is a self-selected sample and no longer an unbiased sample from a randomized trial (see 16-iii).

subitem not at all important

1 ○

2 ○

3 ○

4 ○

5 ○

essential

Does your paper address subitem 18-i?

Copy and paste relevant sections from the manuscript (include quotes in quotation marks "like this" to indicate direct quotes from your manuscript), or elaborate on this item by providing additional information not in the ms, or briefly explain why the item is not applicable/relevant for your study

您的回答

19) All important harms or unintended effects in each group

Does your paper address CONSORT subitem 19? *

Copy and paste relevant sections from the manuscript (include quotes in quotation marks "like this" to indicate direct quotes from your manuscript), or elaborate on this item by providing additional information not in the ms, or briefly explain why the item is not applicable/relevant for your study

No adverse events or unintended effects were observed in either group throughout the study period.

19-i) Include privacy breaches, technical problems

Include privacy breaches, technical problems. This does not only include physical "harm" to participants, but also incidents such as perceived or real privacy breaches [1], technical problems, and other unexpected/unintended incidents. "Unintended effects" also includes unintended positive effects [2].

subitem not at all important

1 ○

2 ○

3 ○

4 ○

5 ○

essential

Does your paper address subitem 19-i?

Copy and paste relevant sections from the manuscript (include quotes in quotation marks "like this" to indicate direct quotes from your manuscript), or elaborate on this item by providing additional information not in the ms, or briefly explain why the item is not applicable/relevant for your study

您的回答

19-ii) Include qualitative feedback from participants or observations from staff/researchers

Include qualitative feedback from participants or observations from staff/researchers, if available, on strengths and shortcomings of the application, especially if they point to unintended/unexpected effects or uses. This includes (if available) reasons for why people did or did not use the application as intended by the developers.

subitem not at all important

1 ○

2 ○

3 ○

4 ○

5 ○

essential

Does your paper address subitem 19-ii?

Copy and paste relevant sections from the manuscript (include quotes in quotation marks "like this" to indicate direct quotes from your manuscript), or elaborate on this item by providing additional information not in the ms, or briefly explain why the item is not applicable/relevant for your study

您的回答

DISCUSSION

22) Interpretation consistent with results, balancing benefits and harms, and considering other relevant evidence

NPT: In addition, take into account the choice of the comparator, lack of or partial blinding, and unequal expertise of care providers or centers in each group

22-i) Restate study questions and summarize the answers suggested by the data, starting with primary outcomes and process outcomes (use)

Restate study questions and summarize the answers suggested by the data, starting with primary outcomes and process outcomes (use).

subitem not at all important

1 ○

2 ○

3 ○

4 ○

5 ○

essential

Does your paper address subitem 22-i? *

Copy and paste relevant sections from the manuscript (include quotes in quotation marks "like this" to indicate direct quotes from your manuscript), or elaborate on this item by providing additional information not in the ms, or briefly explain why the item is not applicable/relevant for your study

Prior studies on HbA1c management have demonstrated that WeChat-delivered intervention tools—validated across 6 RCTs—yield a notable effect size of 0.84 (95% CI: −0.22 to -1.46) [21]. Remarkably, in our MNT intervention cohort, HbA1c levels substantially decreased. This promising outcome is most likely due to the mHealth technology we crafted. ...
Evidence indicates that when HbA1c levels are less than 7.3%, 2hPG may contribute up to 70% of total HbA1c elevation [28]. In our GDM cohort, FPG had a mean of 5.38 mmol/L (SD: 0.69), with an average HbA1c of 7.17% (SD: 0.71%), suggesting that the increase in HbA1c in this population is largely attributable to increased 2hPG levels. ...
Maternal weight gain throughout pregnancy constitutes a fundamental physiological process of gestation. Our regression model revealed a highly significant time main effect (β = 15.10, 95% CI: 13.96–16.25; P < 0.001), indicating that a substantial increase in maternal weight occurs as pregnancy progresses. ...These results align with the conclusions of previous studies, further confirming the potential of mHealth tools as an adjunct to routine antenatal care.

22-ii) Highlight unanswered new questions, suggest future research

Highlight unanswered new questions, suggest future research.

subitem not at all important

1 ○

2 ○

3 ○

4 ○

5 ○

essential

Does your paper address subitem 22-ii?

Copy and paste relevant sections from the manuscript (include quotes in quotation marks "like this" to indicate direct quotes from your manuscript), or elaborate on this item by providing additional information not in the ms, or briefly explain why the item is not applicable/relevant for your study

您的回答

20) Trial limitations, addressing sources of potential bias, imprecision, and, if relevant, multiplicity of analyses

20-i) Typical limitations in ehealth trials

Typical limitations in ehealth trials: Participants in ehealth trials are rarely blinded. Ehealth trials often look at a multiplicity of outcomes, increasing risk for a Type I error. Discuss biases due to non-use of the intervention/usability issues, biases through informed consent procedures, unexpected events.

subitem not at all important

1 ○

2 ○

3 ○

4 ○

5 ○

essential

Does your paper address subitem 20-i? *

Copy and paste relevant sections from the manuscript (include quotes in quotation marks "like this" to indicate direct quotes from your manuscript), or elaborate on this item by providing additional information not in the ms, or briefly explain why the item is not applicable/relevant for your study

This study has several limitations. First, it was impossible to implement blinding for the personnel involved in the WeMNT intervention. To address this issue, we crafted a protocol to guide the intervention, refined the guidance to align with it, and equipped the team with the skills to execute it effectively. Second, this study, which was conducted in a single region with a homogeneous population, has limitations in terms of generalizability. To bridge the implementation gap, we documented each mHealth module's design and implementation, aligned with those of the BCW. This supports cross-setting validation and real-world generalization. Third, our study focused on short-term outcomes; long-term effects on mothers and children remain undetermined. Future research should recruit larger, heterogeneous cohorts to evaluate the efficacy across diverse populations and extended timeframes. Finally, although we adjusted for key covariates in the sensitivity analyses, there may still be unmeasured confounders (e.g., genetic predisposition and family medical history) that could influence the study outcomes. Future research should use multivariate regression or mixed-effects models to achieve more effective control over these factors.

21) Generalisability (external validity, applicability) of the trial findings

NPT: External validity of the trial findings according to the intervention, comparators, patients, and care providers or centers involved in the trial

21-i) Generalizability to other populations

Generalizability to other populations: In particular, discuss generalizability to a general Internet population, outside of a RCT setting, and general patient population, including applicability of the study results for other organizations

subitem not at all important

1 ○

2 ○

3 ○

4 ○

5 ○

essential

Does your paper address subitem 21-i?

Copy and paste relevant sections from the manuscript (include quotes in quotation marks "like this" to indicate direct quotes from your manuscript), or elaborate on this item by providing additional information not in the ms, or briefly explain why the item is not applicable/relevant for your study

您的回答

21-ii) Discuss if there were elements in the RCT that would be different in a routine application setting

Discuss if there were elements in the RCT that would be different in a routine application setting (e.g., prompts/reminders, more human involvement, training sessions or other co-interventions) and what impact the omission of these elements could have on use, adoption, or outcomes if the intervention is applied outside of a RCT setting.

subitem not at all important

1 ○

2 ○

3 ○

4 ○

5 ○

essential

Does your paper address subitem 21-ii?

Copy and paste relevant sections from the manuscript (include quotes in quotation marks "like this" to indicate direct quotes from your manuscript), or elaborate on this item by providing additional information not in the ms, or briefly explain why the item is not applicable/relevant for your study

您的回答

OTHER INFORMATION

23) Registration number and name of trial registry

Does your paper address CONSORT subitem 23? *

Copy and paste relevant sections from the manuscript (include quotes in quotation marks "like this" to indicate direct quotes from your manuscript), or elaborate on this item by providing additional information not in the ms, or briefly explain why the item is not applicable/relevant for your study

Trial Registration: Chinese Clinical Trial Registry ChiCTR2300078227, https://www.chictr.org.cn/showproj.html?proj=199247

24) Where the full trial protocol can be accessed, if available

Does your paper address CONSORT subitem 24? *

Cite a Multimedia Appendix, other reference, or copy and paste relevant sections from the manuscript (include quotes in quotation marks "like this" to indicate direct quotes from your manuscript), or elaborate on this item by providing additional information not in the ms, or briefly explain why the item is not applicable/relevant for your study

The methods section describes the full details of the trial protocol.

25) Sources of funding and other support (such as supply of drugs), role of funders

Does your paper address CONSORT subitem 25? *

Copy and paste relevant sections from the manuscript (include quotes in quotation marks "like this" to indicate direct quotes from your manuscript), or elaborate on this item by providing additional information not in the ms, or briefly explain why the item is not applicable/relevant for your study

This work was supported by Science and Technology Development Program of Jinan Municipal Health Commission (2024205001)

X27) Conflicts of Interest (not a CONSORT item)

X27-i) State the relation of the study team towards the system being evaluated

In addition to the usual declaration of interests (financial or otherwise), also state the relation of the study team towards the system being evaluated, i.e., state if the authors/evaluators are distinct from or identical with the developers/sponsors of the intervention.

subitem not at all important

1 ○

2 ○

3 ○

4 ○

5 ○

essential

Does your paper address subitem X27-i?

Copy and paste relevant sections from the manuscript (include quotes in quotation marks "like this" to indicate direct quotes from your manuscript), or elaborate on this item by providing additional information not in the ms, or briefly explain why the item is not applicable/relevant for your study

The authors declare that they have no competing interests.

About the CONSORT EHEALTH checklist

As a result of using this checklist, did you make changes in your manuscript? *

○ yes, major changes

◉ yes, minor changes

○ no

What were the most important changes you made as a result of using this checklist?

您的回答

How much time did you spend on going through the checklist INCLUDING making changes in your manuscript *

two hours spent on going through the checklist

As a result of using this checklist, do you think your manuscript has improved? *

- (●) yes
- ( ) no
- ( ) 其他:

Would you like to become involved in the CONSORT EHEALTH group?

This would involve for example becoming involved in participating in a workshop and writing an "Explanation and Elaboration" document

- ( ) yes
- (●) no
- ( ) 其他:

Any other comments or questions on CONSORT EHEALTH

您的回答

STOP - Save this form as PDF before you click submit

To generate a record that you filled in this form, we recommend to generate a PDF of this page (on a Mac, simply select "print" and then select "print as PDF") before you submit it.

When you submit your (revised) paper to JMIR, please upload the PDF as supplementary file.

Don't worry if some text in the textboxes is cut off, as we still have the complete information in our database. Thank you!

Final step: Click submit !

Click submit so we have your answers in our database!

此内容不是由 Google 所创建，Google 不对其作任何担保。 - 服务条款 - 隐私权政策

此表单看起来很可疑吗？ 报告

Google 表单
